# Phenotypic and Molecular Characterization of Winter Wheat Genotypes for Grain Yield, Quality Traits, and HMW Glutenin Subunits

**DOI:** 10.3390/plants15192965

**Published:** 2026-09-29

**Authors:** Zafar M. Ziyaev, Ozod S. Turaev, Maftuna B. Sodikova, Abbos B. Elmurodov, Abdumurod E. Hakimov, Saidjon E. Teshaboev, Dilafruz V. Solieva, Saidmurat K. Baboev, Ram C. Sharma

**Affiliations:** 1 Research Institute of Plant Genetic Resources, The National Center for Knowledge and Innovation in Agriculture, Tashkent 111202, Uzbekistan; ozodturaev@gmail.com (O.S.T.); andijanpgri@gmail.xom (S.E.T.); 2 Institute of Genetics and Plant Experimental Biology, The Academy of Sciences of Uzbekistan, Tashkent 111226, Uzbekistan; msodiqova333@gmail.com (M.B.S.); elmurodov.abb@yandex.ru (A.B.E.); xakimovabdumurod148@gmail.com (A.E.H.); sai-baboev@yandex.ru (S.K.B.); 3 Department of Botany and Genetics, National University of Uzbekistan named after Mirzo Ulugbek, Tashkent 100174, Uzbekistan; soliyevadilafruz82@gmail.com; 4 Central Asia and the Caucasus Regional Office, International Center for Agricultural Research in the Dry Areas (ICARDA), 6 Osiyo Street, Tashkent 100084, Uzbekistan

**Keywords:** gluten, glutenin subunits, grain yield, molecular analysis, protein, quality, test weight, wheat

## Abstract

High grain yield (GY) and improved quality, including the presence of high-molecular-weight glutenin subunits (HMW-GS), determine the commercial success of a wheat (*Triticum aestivum* L.) variety. A field study was conducted in 2021 and 2022 in Kibray, Uzbekistan, using 100 winter wheat germplasm to identify superior genotypes for GY and quality-related traits. GGE biplot analysis was conducted to identify superior genotypes. Molecular analysis was conducted using 8 markers to determine allelic variation and the presence of HMW-GS. The wheat genotypes showed arrays of variation for 17 morpho-physiological and grain- and quality-related traits and allelic diversity for HMW-GS. There was a high prevalence (74%) of the “5 + 10” HMW-GS at the “*Glu-D1*” locus, indicating that the 100 genotypes hold significant genetic value for improving flour strength. The five most superior genotypes based on the GGE biplot analysis for higher GY, TKW, TWT, protein, and gluten were #47(20AWYT-IR-87), #30(27FAWWON-IR-142), #6(27FAWWON-IR-31), #42(20AWYT-IR-54), and #3(27FAWWON-IR-13). Among these, genotypes #3, #6, and #42 carried the favorable “5 + 10” HMW-GS combination and had a Payne score of 9 (Good Quality), whereas genotypes #30 and #47 carried the “5 + 12” combination and had a Payne score of 7 (Medium Quality). The novelty of this study lies in the integration of multi-trait phenotypic characterization with HMW-GS molecular profiling, offering a robust and comprehensive approach to identify superior wheat genotypes valuable for breeding programs targeting simultaneously high grain yield and improved bread-making quality.

## 1. Introduction

The development of new varieties with high grain yield and improved end-use and bread-making quality is an important objective of winter wheat (*Triticum aestivum* L.) research programs in Central Asia [1]. However, breeding for high yield and improved quality is challenging due to the strong influence of the environment on these quantitative traits, as reflected by genotype × environment interactions reported in previous studies [1,2]. Also, the negative correlation between grain yield and quality parameters [3,4] is an additional hurdle in developing high grain-yielding winter wheat varieties with improved quality. This has led to the continuous cultivation of older varieties in Central Asia, such as Bezostaya 1, for their superior quality despite lower grain yields and susceptibility to diseases [5]. Therefore, to replace the lower-yielding older varieties, the new varieties must possess not only high grain yield but also improved quality-related traits.

There is a more dominant influence of weather elements on quality-related traits of winter wheat compared to spring wheat [6]. The diverse winter wheat growing environments in Central Asia have been reported in previous studies [2,7]. Also, the presence of significant effects of environment and genotype × environment (GE) interaction on grain yield has been documented in the region [2,7]. This was also supported by the studies conducted in the regions surrounding Central Asia [8]. Besides grain yield, the significant effect of GE interaction on quality-related traits in wheat has been reported in other studies [1,9,10], suggesting challenges in developing winter wheat varieties with high grain yield and improved quality-related traits.

Broadly, the major quality-related traits in winter wheat include 1000-kernel weight (TKW), test weight (TWT), and the protein and gluten content. According to Zhygunov et al. [11], the quality of wheat grain used for bread production is assessed by flour milling (TWT, TKW, grain vitreousness, and grain size) and baking properties (protein content, sedimentation volume, wet gluten content and its quality, physical properties of the dough, and bread volume). Previous studies found a positive correlation of grain yield with TKW [1,4] and with TWT [3] and a weak negative correlation of grain yield with protein content [12]. However, other studies found a non-significant correlation between grain yield and protein content [1,3,4].

Even though protein and wet gluten contents are primarily considered in selecting a wheat variety in developing countries, the strength and elasticity of gluten are the primary factors that determine bread-making quality [13,14]. Glutenin subunits are the individual, smaller protein building blocks that link together to form the large, complex glutenin polymers. Monomeric gliadins and polymeric glutenins are the two main types of gluten proteins found in storage grains. Based on their molecular weights, the latter are further separated into high molecular weight glutenin subunits (HMW-GS) and low molecular weight glutenin subunits (LMW-GS). HMW-GS contribute significantly to the diversity in dough strength and elasticity, although constituting just around 12% of all grain proteins [15].

New winter wheat varieties with higher grain yield and improved quality-related traits are needed to replace the older varieties. While a diverse set of leading cultivars, advanced breeding lines, and old landraces are stored in the genebank of Uzbekistan, their specific HMW-GS profiles and how these molecular features correlate with multi-year phenotypic performance in this region remain largely uncharacterized. Bridging this knowledge gap is essential for targeted breeding. Hence, this study was conducted to integrate multi-trait phenotypic evaluation with HMW-GS molecular profiling among 100 diverse winter wheat genotypes. The specific objectives were to determine genotypic variation for yield and quality traits, identify superior genotypes, and uncover the presence of elite HMW-GS alleles, ultimately providing comprehensive new information to accelerate the development of high-yielding and high-quality wheat varieties.

## 2. Results

### 2.1. Phenotypic Variation

Analyses of variance for individual traits showed significant differences among 100 genotypes for all traits in each year (individual ANOVA not presented). The combined analysis of variance revealed a significant effect of year on all traits except PHT (Table 1). The 100 genotypes differed significantly for each trait. Genotype × year interaction was significant for each trait.

The set of 100 genotypes exhibited variation across all traits reported in this study. The data on mean values for the 17 traits are available online (Supplementary Table S2). Summary of mean and range of values for different traits are presented in Table 2 that include days to heading (154–166 days) and maturity (200–212 days), plant height (79–116 cm), peduncle length (30–46 cm), spike length (7.9–12.3 cm), number of spikelet per spike (15–22), single spike weight (1.8–3.5 g), grain weight per spike (1.4–2.6 g), kernel per spike (36–66), grain yield (4.11–7.45 t/ha), 1000-kernel weight (30–46 g), test weight (665–800 g/L), protein content (13.0–17.2%), gluten content (25.9–36.8%), gluten deformation index (74–114), sedimentation (26–50 mL) and grain vitreousness (56–87%). Figure 1 shows a biplot analysis of genotypic variations among 100 genotypes for 17 traits in a biplot. The 100 genotypes were distributed among the four quadrants without any apparent skewness. Seventeen traits were placed at different points in the biplot with varying vector lengths.

Grain yield showed a significant positive correlation with TWT (r = 0.30; *p* < 0.01), and a significant negative correlation with protein (r = −0.22; *p* < 0.05) and gluten (r = −0.21; *p* < 0.05) contents. Protein content showed a significant positive correlation with gluten content (r = 0.68; *p* < 0.01). Other correlations among grain yield and quality-related traits were nonsignificant. Biplot analysis of the relationship among different traits and the relative importance of different traits for genotypic variations among 100 genotypes is presented in Figure 2. There were four different groups of traits. The relative importance of different traits for genotypic variations is shown by the length of the vectors. Protein content, plant height (PHT), peduncle length (PL), spikelet per spike (SPKLTPS), spike length (SPL), spike weight (SPKWT), and grain weight per spike (GWTPSPK) showed greater variation than other traits among the 100 genotypes.

A list of 10 superior genotypes based on GGE biplot analysis using grain yield and four quality-related traits is given in Table 3. These genotypes are superior based on their closeness to the position of the ideal genotype located at the center of the concentric rings in the biplot (Figure 3).

### 2.2. HMW-GS Allelic Composition and Frequency Analysis

Molecular screening of 100 winter bread wheat genotypes using eight diagnostic markers revealed a high level of allelic variation at the *Glu-1* loci, which are the primary determinants of dough rheological properties. PCR amplification with locus-specific primers successfully identified the targeted subunits, as evidenced by the clear and reproducible banding patterns for the *Glu-A1* marker (Figure 4). Across the 100 genotypes, 16 locus-specific amplicons (bands) were detected alongside three null states (Table 4). These included 12 diagnostic amplicons corresponding to known functional HMW-GS alleles and four uncharacterized or non-target polymorphic bands (400 bp at *Glu-A1*; 760 bp and 810 bp at *Glu-B1*; 625 bp at *Glu-D1*). The distribution of these alleles was significantly non-uniform, reflecting both the selective pressure for quality in modern breeding and the unique genetic background of the RIPGR germplasm (Figure 5).

Genetic diversity was reflected through the mean effective number of alleles (Ne) 1.49 and expected heterozygosity (He) of 0.26 (Table 5). The By9 marker proved to be the most polymorphic tool in this study, with three alleles and a Polymorphic Information Content (PIC) of 0.508.

### 2.3. Evaluation of Technological Quality Potential via Payne Scoring

At the *Glu-A1* locus, the elite 2* subunit (identified by 344 and 1319 bp amplicons) was predominant, appearing in 60.4% of the accessions (Table 6). The *Glu-B1* locus exhibited the highest degree of polymorphism, with five distinct subunit combinations. The 7/17 subunit was the most frequent (47.42%), followed closely by the high-quality “7/17 + 8” combination (41.24%). At the *Glu-D1* locus, the elite “5 + 10” combination was highly dominant, occurring in 74% of the accessions. Integration of the HMW-GS data into the Payne scoring system produced total scores ranging from 6 to 10 (Table 7). Twenty-eight genotypes achieved the maximum observed score of 10 and were classified as Elite Quality, whereas 54 genotypes scored 8–9 (Good Quality) and 18 genotypes scored 6–7 (Medium Quality). All genotypes with the maximum score of 10 carried the “7/17 + 8” combination at the *Glu-B1* locus and the favorable “5 + 10” combination at *Glu-D1*; 17 of these genotypes also possessed the 2 subunit at *Glu-A1*.

### 2.4. Genetic Relationship and Hierarchical Cluster Analysis

To elucidate the genetic relationships among the 100 accessions, a Hierarchical Cluster Analysis (HCA) was performed using the Ward.D2 linkage algorithm and Jaccard distance (Figure 6). The resulting hierarchical dendrogram separated the 100 wheat accessions into four major genetic clusters based on HMW-GS allelic variation.

The spatial distribution of the accessions was further clarified using Principal Component Analysis (PCA) (Figure 7). The first two principal components (PC1 and PC2) explained 41.79% of the cumulative total genetic variance. The PCA plot showed a clear spatial orientation along the PC1 axis, primarily driven by the *Glu-D1* variation (“5 + 10” vs. “5 + 12”).

To test the statistical significance of this population structure, an Analysis of Molecular Variance (AMOVA) was conducted, revealing that 35.04% of the variance resided among clusters, while 64.96% was found within clusters (Table 8).

## 3. Discussion

### 3.1. Phenotypic Analysis

Mean values for 17 traits of the 100 genotypes included in this study differed significantly in both years, suggesting the presence of genotypic variations among them, which is necessary to identify superior genotypes. The presence of arrays of genotypic variations for the traits studied was also evident from the distribution of the 100 genotypes and 17 traits in the GGE biplot (Figure 1). The presence of significant genotype × year interaction for the traits revealed that relative differences among the genotypes for the traits differed between the two years. Previous studies also reported significant genotype × environment interaction for agronomic and quality-related traits in winter wheat in Central Asia [1,7] and elsewhere [3].

All traits contributed to genotypic variation, which was obvious from the GGE biplot showing relationships among the traits (Figure 2). However, different traits discriminated among the genotypes differently, which was obvious from the vector lengths. The traits that discriminated the most among the 100 genotypes were SPKWT, GWTPSPK, KPS, PL, PHT, and Protein content. Protein and gluten content grouped together, which was different from the group of GY, TWT, and KPS. This suggests that these two groups of yield and quality traits were not related. A significant positive correlation between grain yield and TWT suggested that these two traits can be improved simultaneously. A positive correlation between protein and gluten contents suggested that improvement in one trait would also result in improvement in the second trait. However, the presence of a negative correlation of grain yield with protein and gluten contents would create challenges in simultaneously improving grain yield in combination with high protein or gluten content.

Biplot analysis of the 100 genotypes using GY, TKW, TWT, protein and gluten identified superior genotypes (Figure 3, Table 3). The 10 genotypes were closest to the position of the ideal cultivar. Six of the 10 genotypes originated through IWWIP (four in Turkey and two in Morocco), and two originated in the USA (Table 3). The pedigrees of these superior genotypes are diverse, suggesting that intercrossing among them could result in developing further superior genotypes. The genotypes could also be used as parents in the crossing program to improve commercial cultivars.

Both high grain yield and superior quality-related traits are required in a wheat cultivar for its commercial success. The presence of a negative correlation between yield and quality [3,4] makes it challenging to identify genotypes with superiority for these traits together. In our study, the presence of a low-magnitude negative correlation of grain yield with protein and gluten contents, a non-significant correlation with TKW, and a positive correlation with TWT suggests that grain yield and quality-related traits can be improved together with an appropriate selection strategy for multiple traits. This finding agrees with the previously published reports [1,20,21]. Previous studies also reported selection for high grain yield and one or more superior quality parameters [3,4]. However, multiple quality parameters together with agronomic traits were not included in those studies. Our study has included not only grain yield and quality-related traits, but also several agronomic traits, important for acceptability of a wheat cultivar commercially in addition to high yield and quality. The fact that not all genotypes superior for grain yield were necessarily also better for individual quality traits suggests that some compromise would be required in selecting for grain yield and quality simultaneously. This might necessitate selection criteria for high grain yield and improved quality to be flexible to allow balancing modest deficiency in one or more of the quality traits with obvious gain in yield. This strategy should be considered where food security is important; vice versa could be done in case there is a premium price for high quality. This concept of a flexible selection scheme to allow balancing good qualities against moderate defects has been described as intuitive index selection by Simmonds [22].

### 3.2. Molecular Analysis

The presence of arrays of allelic variation at the three *Glu-1* loci, as exhibited by the eight markers, suggested that this set of 100 genotypes was suitable for identification of new bread wheat varieties with desirable HMW-GS. Further, the distribution of these alleles was significantly non-uniform, reflecting both the selective pressure for quality in modern breeding and the unique genetic background of wheat germplasm in the genebank in Uzbekistan. The successful identification of HMW-GS by the locus-specific primers supports previous reports [16,17,18,19]. The four non-target amplicons found in this study could be utilized in future studies on wheat quality targeting HMW-GS. Further, the distribution of these alleles was significantly non-uniform, reflecting both the selective pressure for quality in modern breeding and the unique genetic background of the improved germplasm and Uzbek genebank accessions.

The values of different parameters for assessing genetic diversity, such as the mean effective number of alleles (Ne) and expected heterozygosity (He), indicated a robust genetic base with considerable plasticity. The marker ‘By9’ was the most polymorphic among the 100 genotypes. The value of polymorphic information content (PIC) was high, suggesting the utility of these markers for marker-assisted selection.

The presence of the 2* subunit at the *Glu-1* locus in 60% of genotypes represented dough strength superior to ‘Ax1 or null alleles in bread-making quality [16,17]. The presence of the combination “7/17 + 8” in 41% of the genotypes at the *Glu-B1* locus aligns with the selection trends for improved dough tenacity as discussed by Lee et al. [23]. Schwarz et al. [18] and Butow et al. [24] noted that the selection of the Dx5 allele is essential for achieving maximum gluten elasticity in wheat. Integration of the HMW-GS profiles into the Payne scoring system indicated a generally favorable predicted technological quality of the evaluated germplasm. The maximum observed Payne score was 10 and was recorded in 28 genotypes. All of these genotypes carried the “7/17 + 8” combination at the *Glu-B1* locus and the favorable "5 + 10” combination at *Glu-D1*, while 17 of the 28 genotypes also possessed the 2 subunit at *Glu-A1*. The revised maximum is consistent with the original *Glu-1* quality scoring system, in which the “5 + 10” combination at *Glu-D1* receives 4 points and makes the greatest locus-specific contribution to the total quality score.

The high frequency of the favorable “5 + 10” combination observed in the present germplasm (74%) is particularly noteworthy in the Central Asian context. Husenov et al. [25] reported a remarkably similar frequency of 74.3% for “5 + 10” in Tajik wheat varieties and breeding lines, where relatively high Payne scores were likewise associated with favorable HMW-GS profiles. This regional similarity suggests that favorable *Glu-D1* alleles have been retained at high frequency in Central Asian breeding materials, potentially reflecting historical selection for bread-making quality. Nevertheless, the *Glu-1* quality score should be interpreted as an indicator of technological potential rather than as a direct measure of end-use quality, because the effects of individual HMW-GS alleles may vary with genetic background and environmental conditions.

The complementary application of HCA, PCA, and AMOVA provided a comprehensive understanding of the population structure. While HCA successfully stratified the germplasm into discrete quality-associated clusters, PCA complemented this by revealing the continuous spatial divergence of these groups, demonstrating that the separation was primarily driven by the *Glu-D1* allelic variation (“5 + 10” vs. “5 + 12”). Furthermore, AMOVA statistically validated this structure, confirming that the genetic differentiation between the four HCA-defined clusters was robust and highly significant (*p* < 0.001). This grouping pattern aligns with the results of Sun et al. [26], confirming that HMW-GS markers are sufficient to categorize wheat cultivars into predicted functional quality groups for targeted breeding. These integrated findings support the conclusions of Ragupathy et al. [27] and Lee et al. [23] regarding the exceptional power of diagnostic markers in discriminating quality-related genetic diversity in winter bread wheat collections.

### 3.3. Implications

This study used previously widely utilized technology of field evaluation of wheat germplasm for grain yield and agronomic performance, routine lab quality analysis for quality-related traits, and molecular analysis of HMW-GS. The novelty of this study lies in the set of germplasm used in this study. The germplasm represents diverse genetic backgrounds and origins, making it suitable for wheat breeding programs widely. A large number of germplasm used in this study originated from IWWP had been shared with winter wheat programs across developing and developed countries. Therefore, the information about the genotypes superior for specific traits or a combination of traits is valuable for many wheat breeding programs, particularly in the developing countries, where either resources or facilities are not available for molecular analysis of wheat bread-making quality. Further, the uniqueness of this study lies in the integration of multi-trait phenotypic characterization with HMW-GS molecular profiling. This is a robust and comprehensive approach to identify superior wheat genotypes valuable for breeding programs targeting simultaneously high grain yield and improved bread-making quality.

## 4. Materials and Methods

### 4.1. Phenotypic Study

#### 4.1.1. Plant Materials

A set of 100 winter wheat genotypes differing in their genetic background and origin was included in this study (Supplementary Table S1). The genotypes were obtained from the genebank accession of the Research Institute of Plant Genetic Resources (RIPGR), Kibray, Uzbekistan. This set of materials has originated from various sources, including the International Winter Wheat Improvement Program (IWWIP) and old and new local wheat varieties of Uzbekistan. IWWIP is a joint wheat breeding program of the Government of Turkey, CIMMYT, and ICARDA.

This particular set of germplasm was selected considering the local and international implications of the useful results at the end of the study. The international implication is reflected through the diverse origins of the germplasm, including Bulgaria, Morocco, Russia, Serbia, Turkey, and the USA. Seventeen genotypes from the Uzbekistan genebank enriched the collection. All genotypes included in this study are available for international exchange on request. Agronomic performance of the IWWIP genotypes across locations in different countries is available online (https://www.iwwip.org; accessed on 23 September 2026) and can also be provided on request from the IWWIP.

#### 4.1.2. Field Experiments

The field experiments were conducted at Kibray (41°23′ N and 69°27′ E, 785 masl) in Tashkent district of Uzbekistan in 2020–2021 (2021) and 2021–2022 (2022) winter wheat crop seasons. The experiment was conducted in an Alpha Lattice design in three replications using 3 m^2^ plots. The experiments were managed with optimum agronomic practices under irrigated conditions.

The data were recorded for several agronomic traits, including grain yield-related and quality-related traits. Plant height (PHT), peduncle length (PEDL), spike length (SPKL), number of spikelets per spike (SPKLTPS), number of kernels per spike (KPS), and spike weight (SPKWT) were recorded according to the previously used procedure [28]. One thousand kernel weight (TKW), test weight (TWT), and grain yield (GY) were recorded using the procedure outlined by Baye et al. [29]. The protein content of wheat grain was determined using the method described by Mishra and Lohumi [30]. The wet gluten content of wheat flour was determined as described by Kaushik et al. [31]. Gluten deformation index (GDI) was determined using the procedure described by Rubets et al. [32]. Zeleny sedimentation value was determined according to the method of AACC [33]. Grain vitreousness was determined according to the procedure outlined by Xie et al. [34].

#### 4.1.3. Statistical Analysis

An analysis of variance was calculated using Genstat 23.1 [35], first for each year. After confirming homogeneity of variances [36], a combined analysis of variance was conducted to test the significance of year, genotypes, and genotype × year interaction. Individual year was considered as a unique and random factor, while genotypic effect was analyzed as fixed.

Genotype and genotype × environment (GGE) biplot software Version 7.10 [37,38] was used to determine genotypic variations and to identify superior genotypes using multiple traits (grain yield, TKW, TWT, and protein and gluten). In the GGE biplot analysis, there is an option that permits comparison among genotypes with a reference genotype. This method specifies the position of an “ideal” genotype. A set of concentric circles is created using the ideal genotype at the concentric center. The ideal genotype is used as a reference to rank the other genotypes. A genotype closer to the point of the ideal genotype is considered superior.

GGE biplot analysis was also used to examine relationships among traits. Each trait in the biplot is connected to the origin with a line, called the vector. The cosine of the angle between the vectors of two traits approximates the correlation coefficient between them. Two traits are positively or negatively correlated if the angles between their vectors are <90° or >90°, respectively. The two traits are independent if the angle between them is near 90°. The traits with longer vectors are more discriminative of the genotypes; short vectors are less discriminative.

### 4.2. Molecular Study

#### 4.2.1. Genomic DNA Extraction

Total genomic DNA of each of the 100 wheat genotypes was isolated from the young leaf tissues of 10-day-old seedlings using a modified Cetyltrimethylammonium Bromide (CTAB) protocol [39]. To ensure high-purity templates for PCR amplification, the extraction included an additional purification step to remove polysaccharides and secondary metabolites, which are common inhibitors in cereal DNA extracts. Seedlings were maintained in a growth chamber at a constant temperature to minimize environmental stress during their early development.

The concentration and purity of the genomic DNA were quantified using a VWR SPEC Twin Touch spectrophotometer (Copenhagen Nanosystems A/S, Hørmarken 2, DK-3520 Farum, Denmark). DNA integrity was further verified by electrophoresis on a 0.9% (*w*/*v*) agarose gel stained with ethidium bromide (Sigma-Aldrich, St. Louis, MO, USA). Only the samples showing a high-molecular-weight band with minimal degradation and an A260/A280 absorbance ratio between 1.8 and 2.0 were selected for further analysis. Finally, all DNA templates were normalized to a working concentration of 50 ng/µL, using sterile deionized water.

#### 4.2.2. PCR Amplification and HMW-GS Diagnostic Markers

The allelic compositions at *Glu-A1*, *Glu-B1*, and *Glu-D1* loci were determined using a set of 8 diagnostic molecular markers (Table 4). These markers were selected for their ability to distinguish high-quality subunits from null or low-quality subunits as reported in previous studies. The primers targeted specific fragment sizes, identifying Ax1, Ax2*, and Axnull variants at the Glu-A1 locus, as well as the key subunits at the *Glu-B1* (Bx7, By8, and By9) and *Glu-D1* (Dx2, Dx5, Dx10, and Dx12) loci.

PCR reactions were conducted in a volume of 10 µL, using 4 µM 2 × EasyTaq^®^ PCR SuperMix (Catalog Number: AS111-13, TransBiotech Co., LTD, Zhuhai, China), 0.5 µM final concentration of each forward and reverse primer, 1 µM of genomic DNA template (25 ng/µL), and nuclease-free water up to the final volume. Thermal cycling was performed on a C1000 Touch Thermal Cycler (Bio-Rad, Hercules, CA, USA) with the following parameters: initial denaturation at 94 °C for 5 min; 35 cycles of denaturation at 94 °C for 30 s, annealing at 54–56 °C (optimized for each primer set) for 30 s, and extension at 72 °C for 45 s; followed by a final extension at 72 °C for 10 min. The amplified bands were visually scored as present (1) or absent (0) to construct the binary data matrix.

#### 4.2.3. Electrophoresis

The amplified PCR products were separated by high-resolution electrophoresis to ensure precise band resolution on 2.0% (*w*/*v*) agarose gels in 0.5× TBE buffer solution. The DNA fragments were stained with ethidium bromide and visualized under UV light using an iBright™ CL1500 Imaging System (Invitrogen, Carlsbad, CA, USA).

#### 4.2.4. Statistical Analysis and Genetic Diversity Metrics

The binary matrix (0/1) generated from the HMW-GS diagnostic markers was subjected to comprehensive genetic and multivariate statistical analyses. To evaluate the informativeness of each diagnostic marker, the Polymorphic Information Content (PIC) was calculated according to Bostein et al. [40]:

$$PIC=1-\sum_{i=1}^{n}P_{i}^{2}-\sum_{i=1}^{n-1}\sum_{j=i+1}^{n}2P_{i}^{2}P_{j}^{2}$$

where, *P_i_* represents the frequency of the *i*-th allele at a specific locus, and *n* is the total count of alleles observed.

Genetic diversity parameters were estimated to quantify the level of polymorphism across the 100 bread wheat genotypes. Expected Heterozygosity (He) or Gene Diversity [41] was calculated as:

$$He=1-\sum_{i=1}^{k}P_{i}^{2}$$

where *P_i_* is the frequency of the *i*-th allele, and *k* is the total number of alleles at the given locus.

The Effective Number of Alleles (Ne) [42] was determined to represent the number of equally frequent alleles required to achieve the same degree of gene diversity:

$$N_{e}\;=\;\frac{1}{\sum_{i=1}^{k}P_{i}^{2}}\;=\;\frac{1}{1-H_{e}}$$

To measure overall genetic diversity and evenness, Shannon’s Information Index (I) [43] was computed as follows:

$$I\;=\;-\;\sum_{i\;=\;1}^{k}P_{i}ln(P_{i})$$

The genetic relationships among accessions were determined using Hierarchical Cluster Analysis (HCA). A dissimilarity matrix was constructed using the Jaccard distance (D_J_):

$$D_{J}=\;1-\;\frac{a}{a\;+\;b\;+\;c}$$

where *a* is the number of shared positive bands (1-1) between two accessions, *b* is the number of bands present only in the first accession, and *c* is the number of bands present only in the second accession. Clustering was performed using the Ward.D2 linkage algorithm to minimize intra-cluster variance.

These three multivariate approaches were employed complementarily to address specific statistical questions: HCA was used to identify discrete genetic groupings (clusters) based on shared allelic states using the Ward.D2 linkage algorithm and Jaccard distance. To complement this, Principal Component Analysis (PCA) was applied to reduce dimensionality, visualize the continuous spatial distribution of these accessions, and identify the primary allelic drivers of genetic variance. Finally, to test the statistical significance of the population structure defined by the HCA, we conducted an AMOVA (with 999 permutations) to partition genetic variance between and within these clusters. All statistical analyses and graphical visualizations were performed in R (v.4.3.x) using the vegan, poppr, FactoMineR, factoextra, and ggrepel packages.

#### 4.2.5. Evaluation of Bread-Making Quality Potential (Payne Score)

To evaluate the technological quality potential of the wheat accessions, the total Payne *Glu-1* Quality Score was assigned to each genotype based on its HMW-GS composition across the *Glu-A1*, *Glu-B1*, and *Glu-D1* loci [44]. Individual scores ranging from 1 to 4 were attributed to specific subunit variants based on their established contributions to dough strength and gluten elasticity. At the *Glu-A1* locus, subunits Ax1 and Ax2* received 3 points each, while the null allele received 1 point. For *Glu-B1*, subunits Bx7 + By8 and Bx7 + By9 were assigned 3 and 2 points, respectively, whereas Bx6 + By8 received 1 point and single Bx7/17 received 2 points. At the *Glu-D1* locus, the favorable Dx5 + Dy10 combination received the highest locus-specific score of 4 points, whereas Dx2 + Dy12 and single Dx5 received 2 points each. The total Payne Score (*S_total_*) for each accession was calculated as the sum of the individual scores across all three loci. The theoretical total score ranges from 3 to 10, whereas the observed scores in the present germplasm ranged from 6 to 10.

The total Payne Score (*S_total_*) for each accession was calculated as the sum of the individual scores across all three loci:

$$S_{total}=\;S_{Glu\;-\;A1}+S_{Glu\;-\;B1}+S_{Glu\;-\;D1}$$

Based on the cumulative Payne scores, genotypes were classified into four functional quality categories: Low Quality (S ≤ 5), Medium Quality (6 ≤ S ≤ 7), Good Quality (8 ≤ S ≤ 9), and Elite Quality (S = 10). In the present germplasm, the observed total Payne scores ranged from 6 to 10.

## 5. Conclusions

Both yield and quality of bread wheat are important to meet food and nutrition security of the growing population in Central Asia. This study confirms the complex effect of genotype × environment interactions on grain yield and quality-related traits. In the past, wheat breeders have found it difficult to combine high yield and quality under diverse environmental conditions, with limited success reported [1]. Our study shows that progress has been made in this direction in developing improved germplasm of winter wheat by the International Winter Wheat Improvement Program. Our study has successfully identified a number of superior genotypes with higher yield in combination with improved quality. Several superior genotypes also possessed favorable HMW-GS combinations and showed moderate-to-high Payne scores, indicating favorable predicted technological potential. However, these molecular-based scores should be considered as indicators of predicted quality potential and not as substitutes for direct dough rheological or bread-making quality measurements. Such superior genotypes could be considered as promising candidates for further multi-environment evaluation and as potential parents in wheat breeding programs. A few superior genotypes for yield and quality, identified in this study, originate from the International Winter Wheat Improvement Program and are distributed to the countries in Central Asia and other regions. Therefore, the findings of this study are valuable for national and international winter wheat breeding programs.

## Figures and Tables

**Figure 1:**
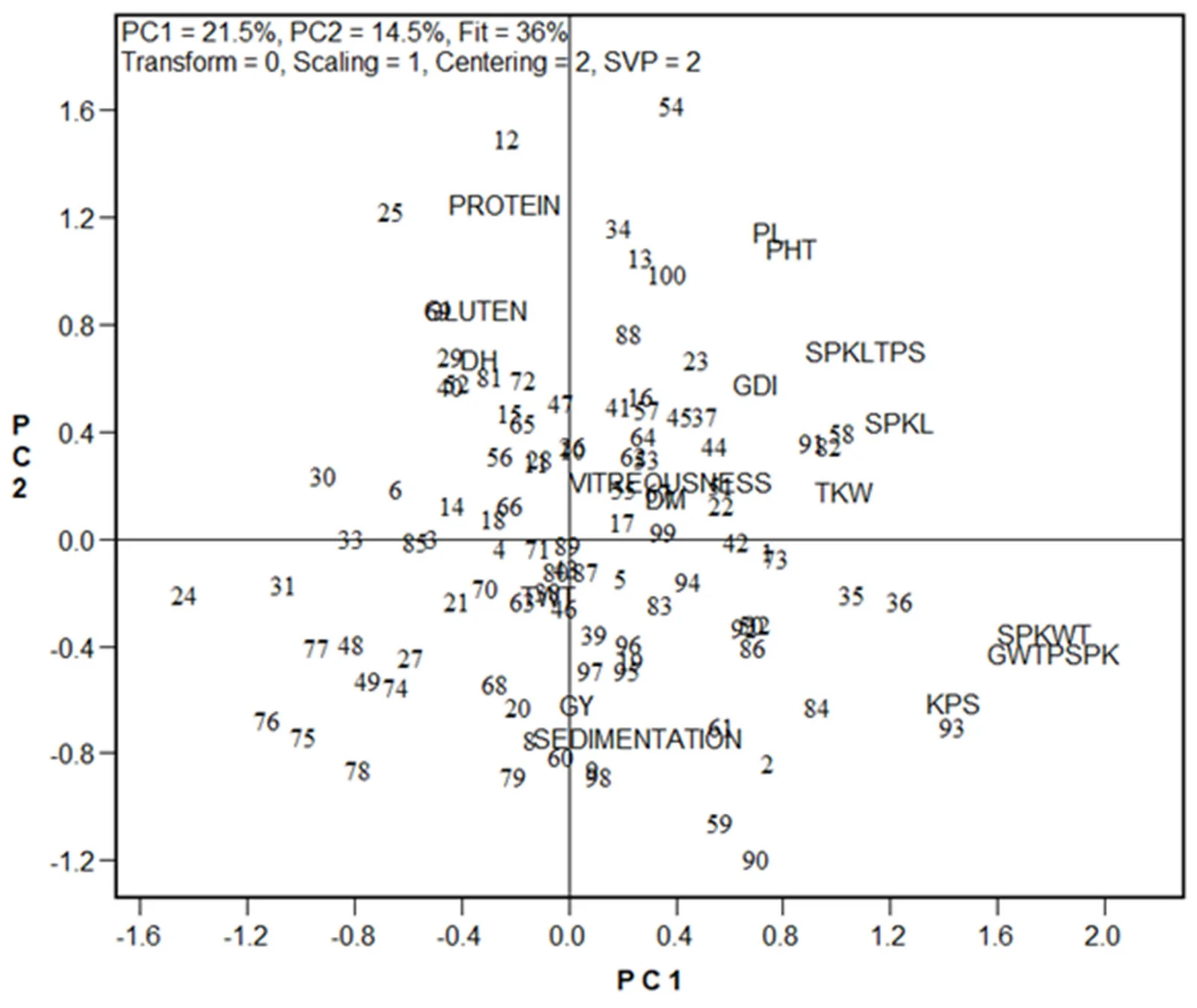
GGE biplot for 17 traits of 100 winter wheat genotypes evaluated in 2021 and 2022 in Kibray, Uzbekistan. The abbreviated names are for the traits. The numbers represent genotypes (Supplementary Table S1). DH = Days to heading, DM = Days to maturity, PHT = Plant height, PL = Peduncle length, SPKL = Spike length, SPKLTPS = Spikelets per spike, SPKWT = Single spike weight, GWTPSPK = Grain weight per spike, KPS = Kernels per spike, GY = Grain yield, TKW = 1000-kernel weight, TWT = Test weight, GDI = Gluten deformation index.

**Figure 2:**
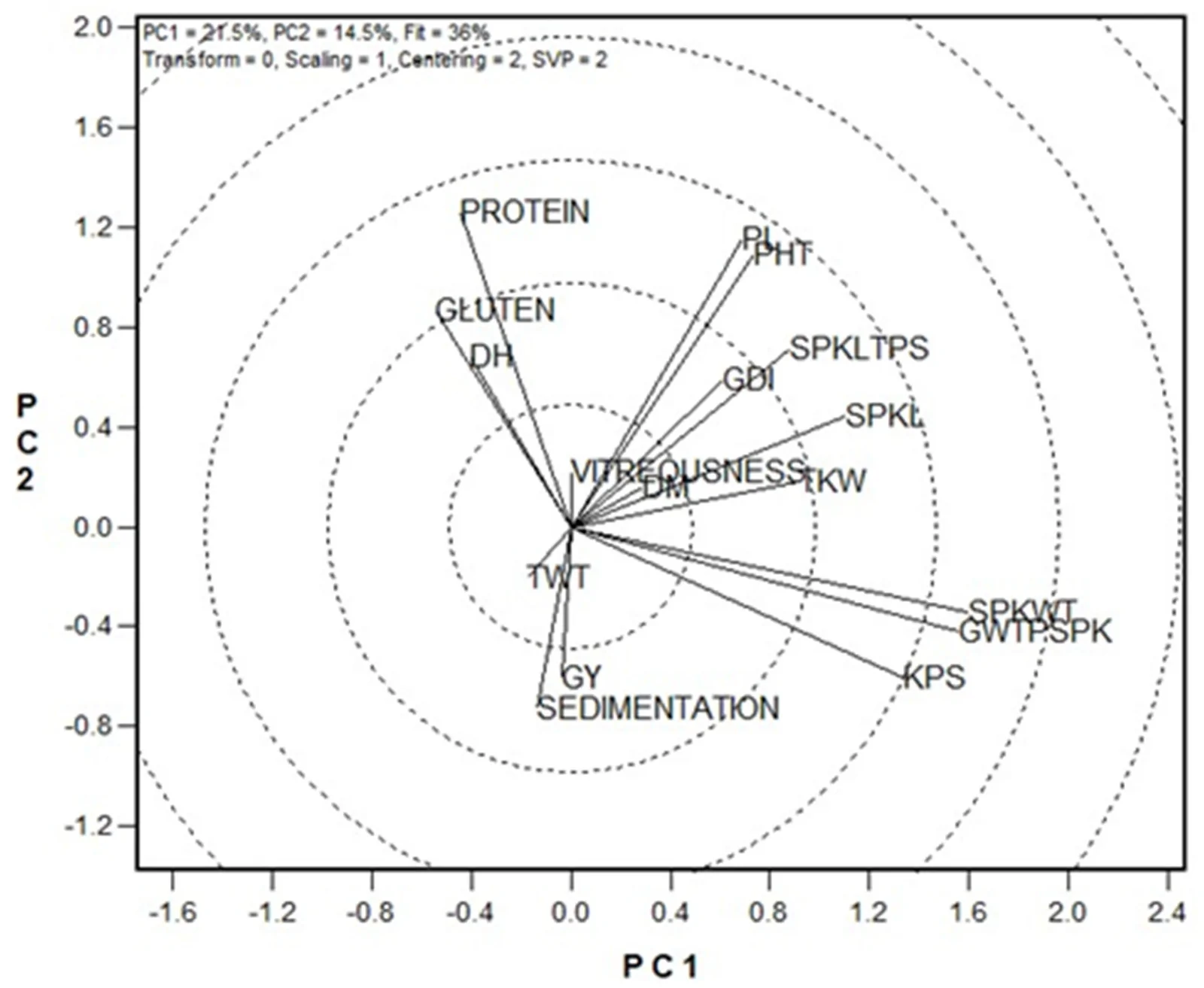
GGE biplot showing the relationship among traits and length of vectors representing importance of a trait for genotypic variations among 100 genotypes. For clarity of the graph, the genotypes are not shown in the biplot. Full names of the abbreviated traits are given in Figure 1.

**Figure 3:**
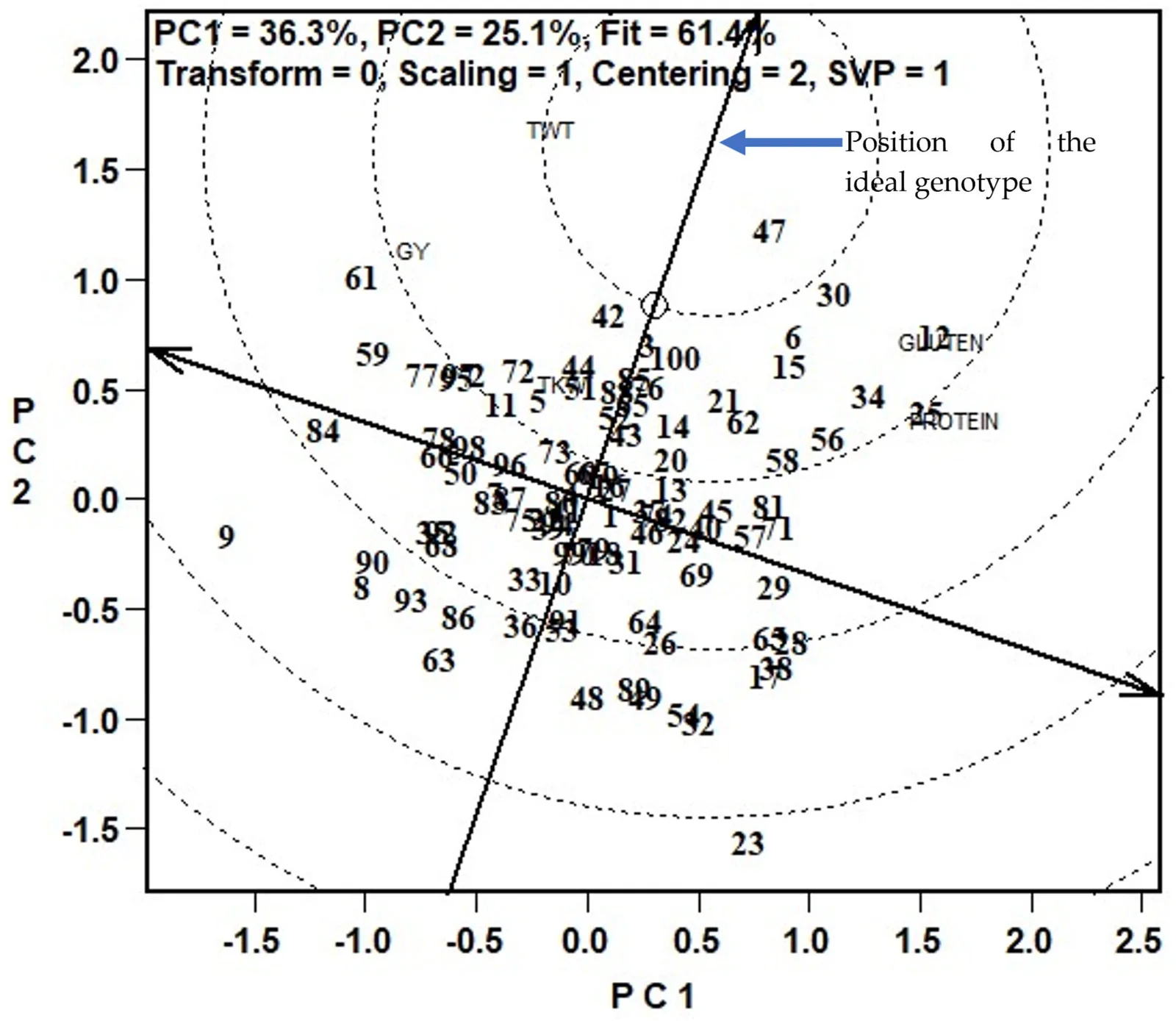
GGE Biplot showing ranking of genotypes relative to an “ideal” genotype based on grain yield (GY), 1000-kernel weight (TKW), test weight (TWT), and protein and gluten contents of 100 winter wheat genotypes (full names are given in Supplementary Table S1) evaluated in two years (2021 and 2022) in Kibray, Uzbekistan.

**Figure 4:**
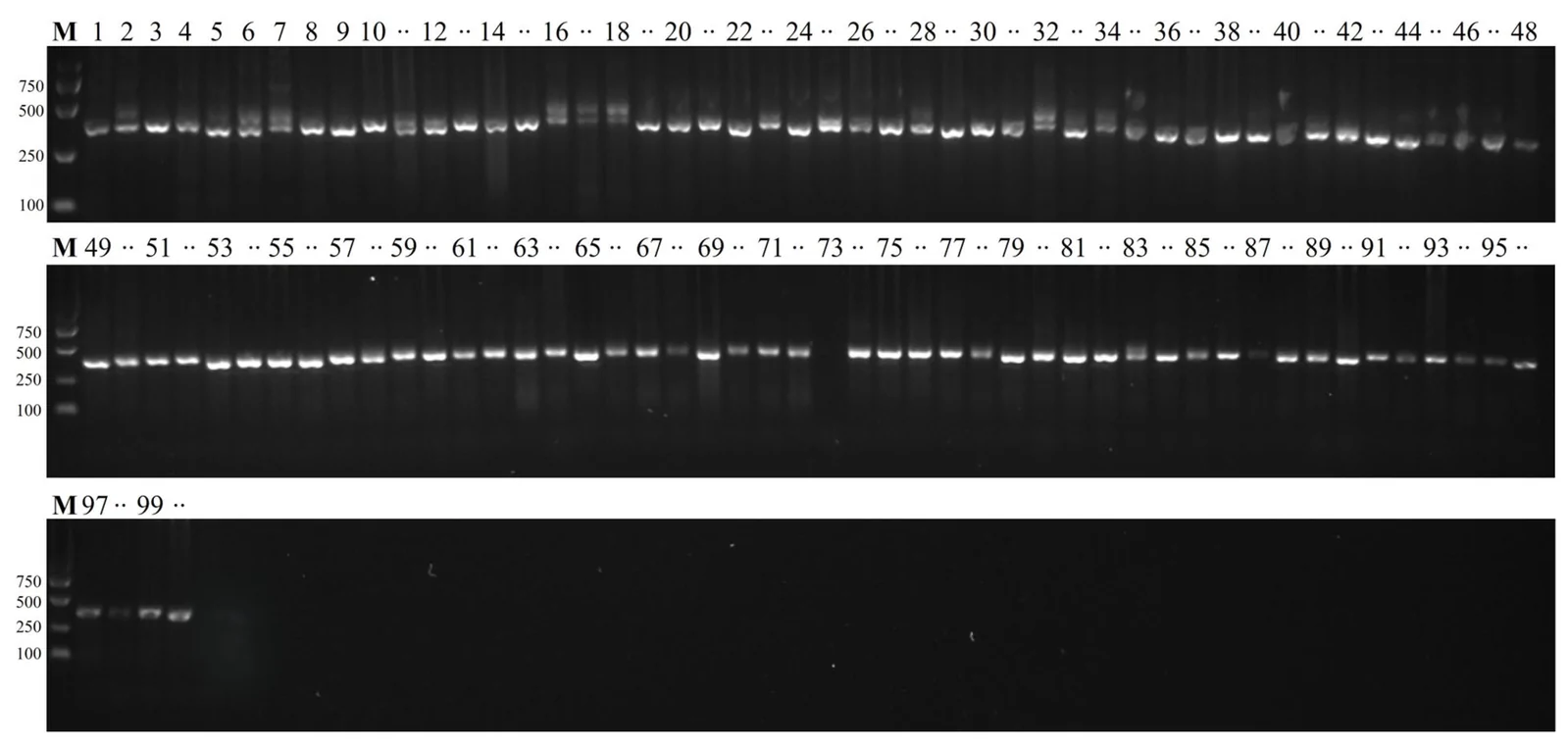
An illustration of gel electrophoresis image showing PCR amplification of the Ax1/Axnull marker at the *Glu-A1* locus for 100 wheat genotypes.

**Figure 5:**
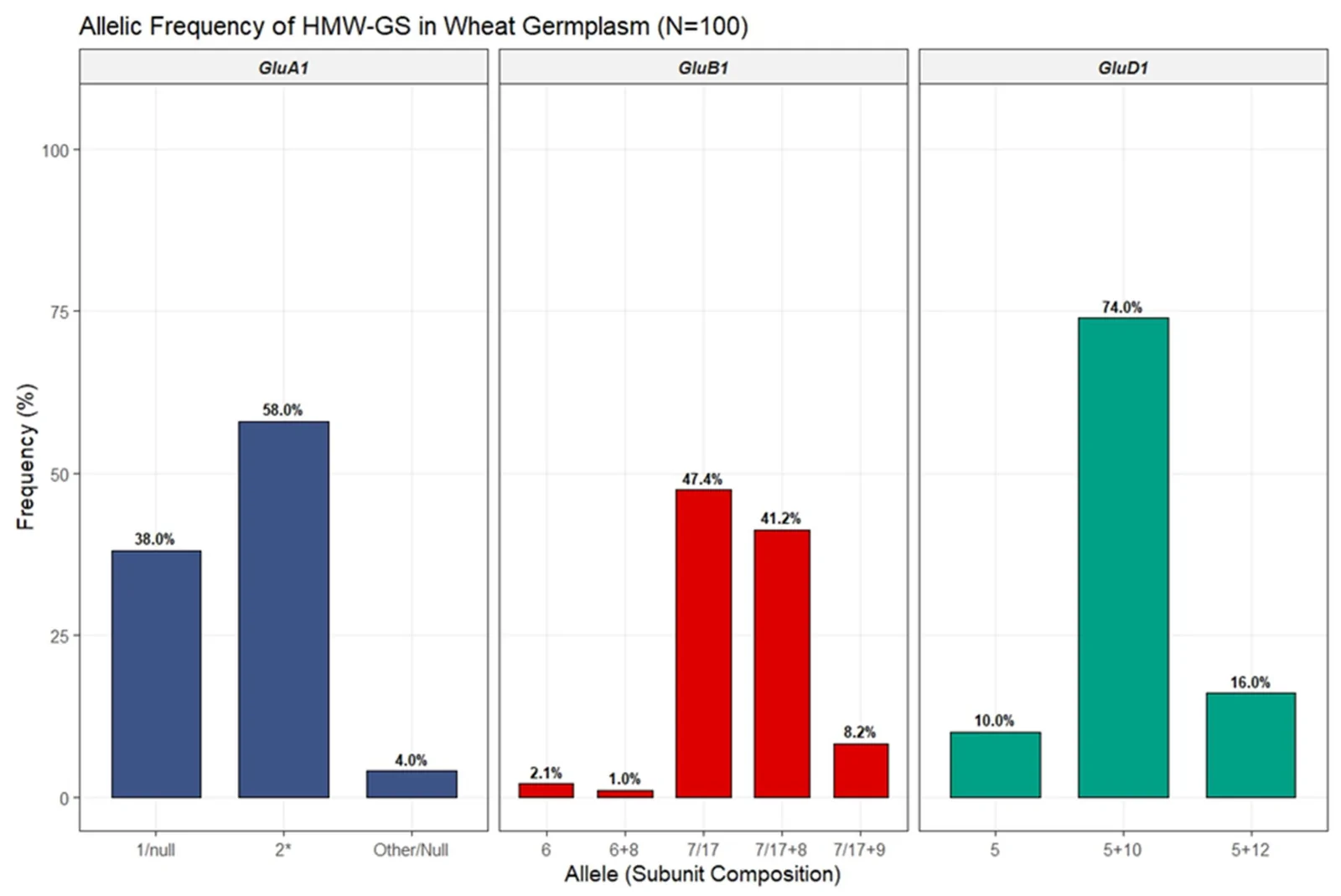
Allelic frequencies of HMW-GS detected at *Glu-A1*, *Glu-B1*, and *Glu-D1* loci using 8 linked markers in 100 wheat genotypes.

**Figure 6:**
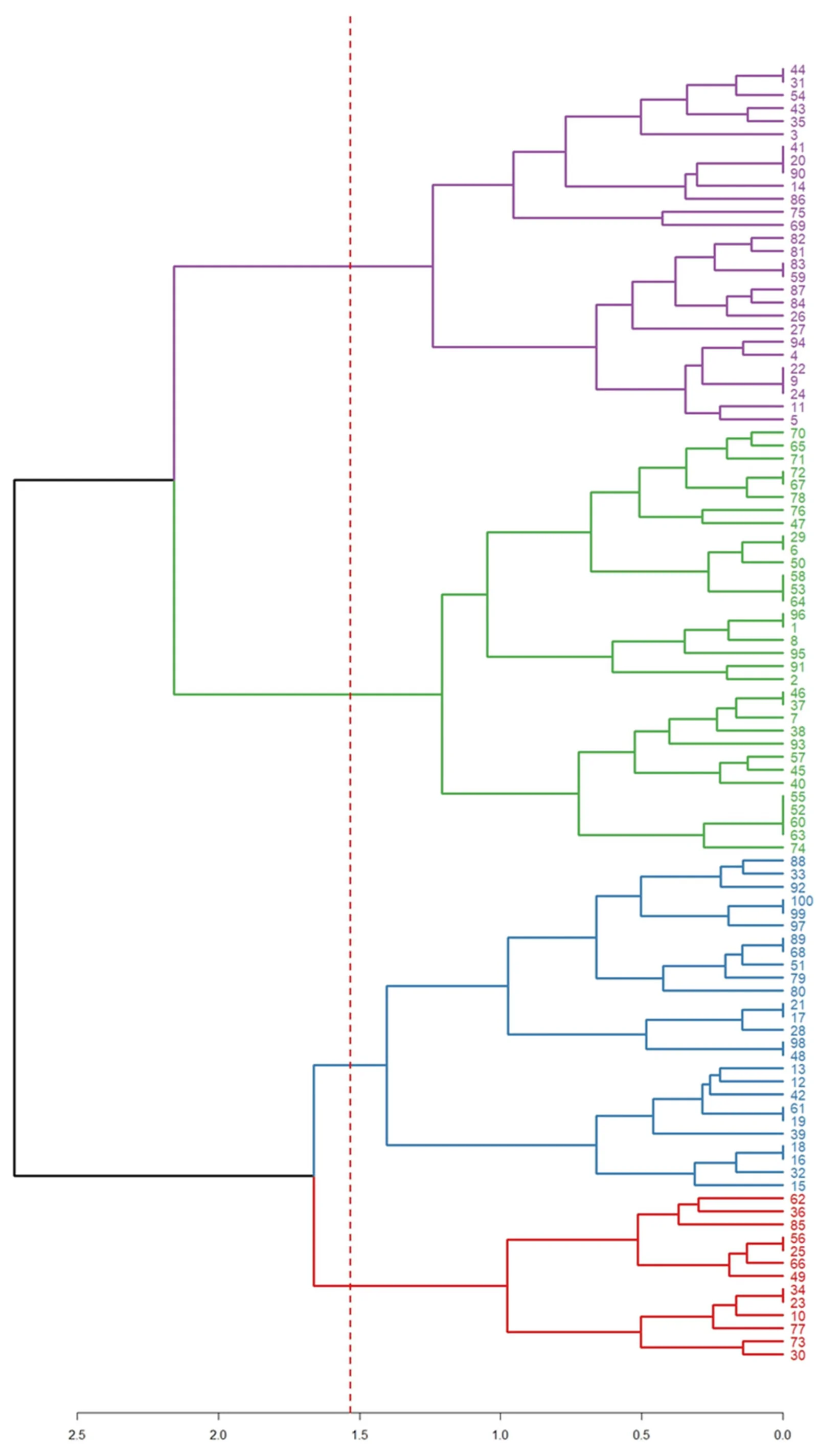
Dendrogram illustrating the genetic relationships of 100 bread wheat accessions based on HMW-GS allelic variation, generated via Hierarchical Cluster Analysis (Ward.D2 linkage and Jaccard distance) using 8 associated polymorphic markers at the *Glu-1* locus.

**Figure 7:**
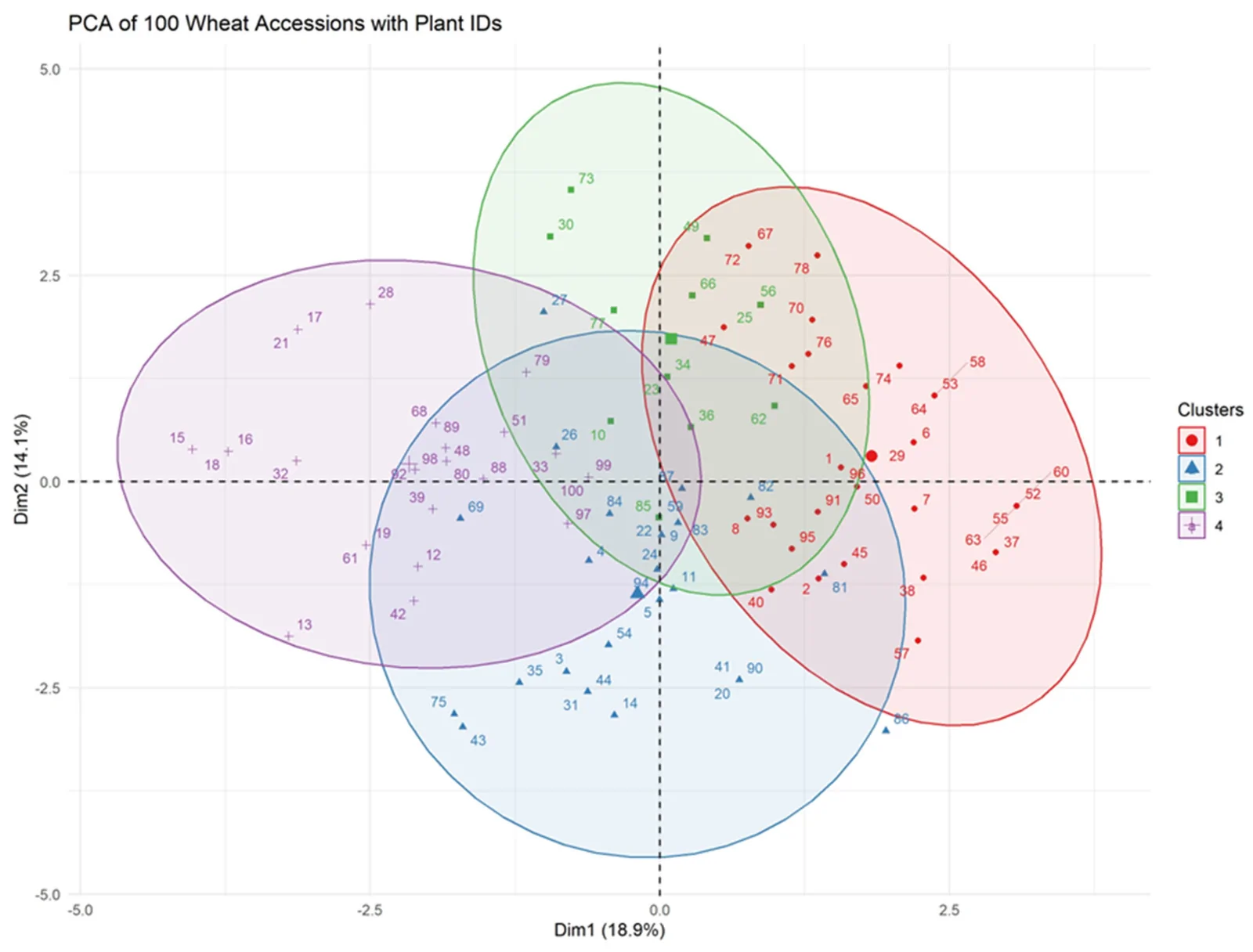
Principal component analysis (PCA) based on 8 linked polymorphic markers at the *Glu-1* locus in 100 wheat genotypes.

**Table 1:** Analysis of variance for various traits of 100 wheat genotypes evaluated in 2021 and 2022 in Kibray, Uzbekistan.

Traits	Year	Rep/Year	Block/Rep Year	Genotype	Geno × Year	Error
	df = 1	df = 4	df = 54	df = 99	df = 99	df = 342
Days to heading	30,232 **	12	9	35 **	11 **	2
Days to maturity	90,454 **	5	12	23 **	15 **	2
Plant height	110	284	90	284 **	56 **	36
Peduncle length	85 **	21	15	60 **	15 **	8
Spike length	3 **	0.17	1.83	4.32 **	1.77 **	0.39
Spikelet per spike	324 **	6	272	963 **	389 **	497
Spike weight	91 **	0.02	0.2	0.64 **	0.33 **	0.02
Grain weight per spike	62 **	0.01	0.12	0.37 **	0.21 **	0.01
Kernel per spike	3455 **	7	42	139 **	67 **	2
Grain yield	11 **	0.08	1.03	2.70 **	0.36 **	0.03
1000-kernel weight	69 **	3.77	14.85	49.88 **	29.62 **	0.61
Test weight	23,802 **	624	1373	3087 **	1905 **	98
Protein content	70 **	1.12	1.23	3.22 **	1.18 **	0.23
Gluten content	93 **	15	12	25 **	13 **	4
Gluten deformation	15,101 **	437	137	375 **	171 **	22
Sedimentation	412 **	8	36	122 **	21 **	4
Vitreous	1815 **	76	64	277 **	99 **	11

** Significant at *p* = 0.01.

**Table 2:** Mean and range of values for different traits in 100 winter wheat genotypes studied in field trials in Kibray, Uzbekistan in two years (2021–2022).

Parameter		Minimum	Maximum	Mean	LSD_0.05_	CV (%)
DH ^#^	days	154	166	161	1.5	1
DM	days	200	212	206	1.7	1
PHT	cm	79	116	100	6.9	6
PL	cm	30	46	37	3	8
SPKL	cm	8	12	10	1	6
SPKLTPS	per spike	15	22	18	1.3	7
SPKWT	g	1.8	3.5	2.52	0.15	6
GWTPSPK	g	1.4	2.6	1.96	0.09	4
KPS	per spike	36	66	48	1.8	3
GY	t/ha	4.11	7.45	5.81	0.2	3
TKW	g	30	46	36.9	0.9	2
TWT	g/L	665	800	756	11	1
Protein ^#^	%	13	17.2	15.1	0.5	3
Gluten ^£^	%	25.9	36.8	31	2.2	6
GDI ^¥^	%	74	114	88.8	5.4	5
Sedimentation ^¶^	ml	26	50	38.3	2.3	5
Vitreousness ^≠^	%	56	87	69	3.8	5

^#^ DH = Days to heading, DM = Days to maturity, PHT = Plant height, PL = Peduncle length, SPKL = Spike length, SPKLTPS = Spikelet per spike, SPKWT = Single spike weight, GWTPSPK = Grain weight per spike, KPS = Kernel per spike, GY = Grain yield, TKW = 1000-kernel weight, TWT = Test weight, GDI = Gluten deformation index. ^#^, ^£^ Minimum values for Grade I and II wheat for Uzbekistan are, respectively, 14 and 12.5% protein and 32 and 28% gluten. ^¥^ GDI value weak, good, satisfactory, and unsatisfactory wheats are <40, 45 to 75, 80 to100, and >105, respectively. ^¶^ Sedimentation of different grades of wheat are poor <20 mL, satisfactory 20 to 30 mL, good 31 to 50 mL and very good >50 mL. ^≠^ Vitreous value for Grade I and II wheat in Uzbekistan is >60%.

**Table 3:** List of 25 superior genotypes identified using GGE biplot for grain yield, 1000-kernel weight, test weight, and protein and gluten contents from among 100 genotypes evaluated in 2021 and 2022 in Kibray, Uzbekistan.

Genotype Number	GGE Rank	Distance to Ideal Cultivar	Selection Number	Pedigree	Origin
47	1	0.4	20AWYT-IR-87	Shi#4414/Crows”//GK Sagvari/CA8055/6/4Won-Ir-257/5/Ymh/Hys//Hys/Tur3055/3/Dga/4/Vpm/Mos	IWWIP-Morocco
30	2	0.8	27FAWWON-IR-142	KS13DH002722	USA-KSU
6	3	0.9	27FAWWON-IR-31	00*0100-51/3/*T. dicoccon* PI94625/*Ae. squarrosa*(372)//3* Pastor	IWWIP-Turkey
42	4	0.9	20AWYT-IR-54	Cadet/6/Yumai13/5/Nai60/3/14.53/Odin//CI13441/Canon/7/Zander//Attila/3*Bcn	IWWIP-Morocco
3	5	0.9	27FAWWON-IR-13	UN-9/6/Attila/*Bcn//Bav92/3/Tilhi/5/Bav92/3/Prl/Sara//Tsi/Vee#5/4/Croc_1/*Ae. squarrosa*(224)//2*Opata	IWWIP-Turkey
100	6	0.9	Pahlavon	Commercial cultivar (Sanzar85/K95137)	Uzbekistan
15	7	1	27FAWWON-IR-70	Aisberg/*Ae. squarrosa*(369)//Demir	IWWIP-Turkey
85	8	1.1	Zvezda	Commercial cultivar (Pedigree unknown)	Uzbekistan
21	9	1.1	27FAWWON-IR-86	Tirchmir1//71ST2959/Crow/3/KS 92 WGRC24/4/Whear/Sokoll	IWWIP-Turkey
76	10	1.1	23IWWYT-IR-9843	KS031009K-4/KS060285-M-1	USA-KSU

**Table 4:** Panel of DNA markers associated with HMW-GS genes.

No.	Primer Name	Primer Sequence (5′–3′)	Subunit	Associated Allele, bp	Detected Amplicon Size, bp	Reference
*Glu-A1*						
1	Ax1/Anull, Ax2*	F: CGAGACAATATGAGCAGCAAG R: CTGCCATGGAGAAGTTGGA	Ax1/Axnull	362	362	[16]
			-	-	400 ^†^	
			Ax2*	344	344	
2	Ax2*	F: ATGACTAAGCGGTTGGTTCTT R: ACCTTGCTCCCCTTGTCTTT		1319	1319	[17]
*Glu-B1*						
3	Bx6, Bx7/Bx17	F: CACTGAGATGGCTAAGCGCC R: GCCTTGGACGGCACCACAGG	Bx6	246	246	[18]
			Bx7/Bx17	231	231	
4	By8	F: TTAGCGCTAAGTGCCGTCT R: TTGTCCTATTTGCTGCCCTT	By8	527	527	[19]
5	By9	F: TTCTCTGCATCAGTCAGGA R: AGAGAAGCTGTGTAATGCC	By9	662	662	
			-	-	760 ^†^	
			-	-	810 ^†^	
*Glu-D1*						
6	Dx2, Dx5	F: GGGACAATACGAGCAGCAAA R: CTTGTTCCGGTTGTTGCCA	Dx2	299	299	[16]
			Dx5	281	281	
7	Dx5	F: CGTCCCTATAAAAGCCTAGC R: AGTATGAAACCTGCTGCGGAC		478	478	[17]
			-		625 ^†^	
8	Dx10, Dx12	F: CGCAAGACAATATGAGCAAACT R: TTGCCTTTGTCCTGTGTGC	Dx10	397	397	[16]
			Dx12	415	415	

^†^ uncharacterized polymorphic bands.

**Table 5:** Genetic diversity indices and allelic frequencies.

Marker_System	Alleles	Expected Heterozygosity (He)	Effective Alleles (Ne)	Shannon Index (I)	PIC
Ax1/Axnull, Ax2*	3	0.4046	1.6795	1.0672	0.18
Ax2*	1	0	1	0	0
Bx6, Bx7/Bx17	2	0.1064	1.1191	0.2884	0.0887
By8	1	0	1	0	0
By9	3	0.5613	2.2795	0.9138	0.5098
Dx2, Dx5	2	0.5244	2.1026	0.6733	0.4412
Dx5	2	0.019	1.0194	0.1151	0.0172
Dx10, Dx12	2	0.4268	1.7446	0.516	0.3988

**Table 6:** Allelic frequencies and subunit distribution of HMW-GS across the *Glu-1* loci in 100 winter wheat genotypes studied for their yield and quality in 2021 and 2022 in Kibray, Uzbekistan.

Genotype ID	*GluA1*	Bx_Unit	By_Unit	*GluB1*	Dx_Unit	Dy_Unit	*GluD1*
1	2*	7/17	null	7/17	5	10	5 + 10
2	2*	null	null	null	5	10	5 + 10
3	1/null	null	null	null	5	10	5 + 10
4	1/null	7/17	null	7/17	5	10	5 + 10
5	2*	7/17	null	7/17	5	10	5 + 10
6	2*	7/17	null	7/17	5	10	5 + 10
7	2*	7/17	null	7/17	5	10	5 + 10
8	null	7/17	null	7/17	5	10	5 + 10
9	2*	7/17	null	7/17	5	10	5 + 10
10	2*	7/17	null	7/17	5	10	5 + 10
11	2*	7/17	null	7/17	5	10	5 + 10
12	2*	7/17	null	7/17	5	10	5 + 10
13	1/null	7/17	null	7/17	5	10	5 + 10
14	1/null	7/17	9	7/17 + 9	5	10	5 + 10
15	1/null	7/17	8	7/17 + 8	5	12	5 + 12
16	1/null	7/17	null	7/17	5	12	5 + 12
17	1/null	7/17	null	7/17	5	12	5 + 12
18	1/null	7/17	null	7/17	5	12	5 + 12
19	1/null	7/17	8	7/17 + 8	5	10	5 + 10
20	1/null	7/17	9	7/17 + 9	5	10	5 + 10
21	1/null	7/17	null	7/17	5	12	5 + 12
22	2*	7/17	null	7/17	5	10	5 + 10
23	2*	7/17	null	7/17	5	10	5 + 10
24	2*	7/17	null	7/17	5	10	5 + 10
25	2*	7/17	8	7/17 + 8	5	10	5 + 10
26	1/null	7/17	8	7/17 + 8	5	null	5
27	2*	7/17	8	7/17 + 8	5	12	5 + 12
28	2*	7/17	null	7/17	5	12	5 + 12
29	2*	7/17	null	7/17	5	10	5 + 10
30	2*	7/17	null	7/17	5	12	5 + 12
31	1/null	7/17	null	7/17	5	10	5 + 10
32	1/null	7/17	null	7/17	5	12	5 + 12
33	2*	7/17	null	7/17	5	10	5 + 10
34	2*	7/17	null	7/17	5	10	5 + 10
35	1/null	7/17	null	7/17	5	10	5 + 10
36	2*	7/17	8	7/17 + 8	5	10	5 + 10
37	2*	7/17	9	7/17 + 9	5	10	5 + 10
38	2*	7/17	9	7/17 + 9	5	10	5 + 10
39	2*	7/17	null	7/17	5	null	5
40	2*	7/17	null	7/17	5	10	5 + 10
41	1/null	7/17	9	7/17 + 9	5	10	5 + 10
42	1/null	7/17	null	7/17	5	10	5 + 10
43	1/null	7/17	null	7/17	5	10	5 + 10
44	1/null	7/17	null	7/17	5	10	5 + 10
45	2*	7/17	null	7/17	5	10	5 + 10
46	2*	7/17	9	7/17 + 9	5	10	5 + 10
47	2*	7/17	null	7/17	5	12	5 + 12
48	1/null	7/17	null	7/17	5	Null	5
49	2*	7/17	8	7/17 + 8	5	Null	5
50	2*	7/17	null	7/17	5	10	5 + 10
51	1/null	7/17	8	7/17 + 8	5	10	5 + 10
52	2*	7/17	8	7/17 + 8	5	10	5 + 10
53	2*	7/17	8	7/17 + 8	5	10	5 + 10
54	1/null	7/17	8	7/17 + 8	5	10	5 + 10
55	2*	7/17	8	7/17 + 8	5	10	5 + 10
56	2*	7/17	8	7/17 + 8	5	10	5 + 10
57	2*	7/17	null	7/17	5	10	5 + 10
58	2*	7/17	8	7/17 + 8	5	10	5 + 10
59	1/null	7/17	8	7/17 + 8	5	10	5 + 10
60	2*	7/17	8	7/17 + 8	5	10	5 + 10
61	1/null	7/17	8	7/17 + 8	5	10	5 + 10
62	2*	7/17	8	7/17 + 8	5	10	5 + 10
63	2*	7/17	8	7/17 + 8	5	10	5 + 10
64	2*	7/17	8	7/17 + 8	5	10	5 + 10
65	2*	7/17	8	7/17 + 8	5	10	5 + 10
66	2*	7/17	8	7/17 + 8	5	10	5 + 10
67	2*	7/17	8	7/17 + 8	5	12	5 + 12
68	1/null	7/17	8	7/17 + 8	5	10	5 + 10
69	1/null	6	null	6	5	12	5 + 12
70	2*	7/17	8	7/17 + 8	5	null	5
71	2*	7/17	null	7/17	5	null	5
72	2*	7/17	8	7/17 + 8	5	12	5 + 12
73	2*	7/17	8	7/17 + 8	5	12	5 + 12
74	2*	7/17	8	7/17 + 8	5	12	5 + 12
75	1/null	6	null	6	5	Null	5
76	2*	7/17	8	7/17 + 8	5	Null	5
77	2*	7/17	null	7/17	5	Null	5
78	2*	7/17	8	7/17 + 8	5	12	5 + 12
79	null	7/17	8	7/17 + 8	5	10	5 + 10
80	null	6	8	6 + 8	5	10	5 + 10
81	2*	7/17	8	7/17 + 8	5	10	5 + 10
82	2*	7/17	8	7/17 + 8	5	10	5 + 10
83	1/null	7/17	8	7/17 + 8	5	10	5 + 10
84	1/null	7/17	8	7/17 + 8	5	10	5 + 10
85	2*	7/17	8	7/17 + 8	5	10	5 + 10
86	2*	7/17	9	7/17 + 9	5	10	5 + 10
87	2*	7/17	8	7/17 + 8	5	10	5 + 10
88	1/null	7/17	null	7/17	5	10	5 + 10
89	1/null	7/17	8	7/17 + 8	5	10	5 + 10
90	1/null	7/17	9	7/17 + 9	5	10	5 + 10
91	2*	null	null	null	5	10	5 + 10
92	1/null	7/17	null	7/17	5	10	5 + 10
93	2*	7/17	null	7/17	5	10	5 + 10
94	1/null	7/17	null	7/17	5	10	5 + 10
95	null	7/17	null	7/17	5	10	5 + 10
96	2*	7/17	null	7/17	5	10	5 + 10
97	1/null	7/17	null	7/17	5	10	5 + 10
98	1/null	7/17	null	7/17	5	null	5
99	1/null	7/17	8	7/17 + 8	5	10	5 + 10
100	1/null	7/17	8	7/17 + 8	5	10	5 + 10

**Table 7:** Distribution of Payne quality scores in 100 wheat genotypes based on molecular analysis of high molecular weight glutenin subunits using 8 markers linked to *Glu-1* locus.

Genotype ID	Score_A1	Score_B1	Score_D1	Total Payne Score	Quality Class
1	3	2	4	9	Good Quality
2	3	2	4	9	Good Quality
3	3	2	4	9	Good Quality
4	3	2	4	9	Good Quality
5	3	2	4	9	Good Quality
6	3	2	4	9	Good Quality
7	3	2	4	9	Good Quality
8	1	2	4	7	Medium Quality
9	3	2	4	9	Good Quality
10	3	1	4	8	Good Quality
11	3	1	4	8	Good Quality
12	3	2	4	9	Good Quality
13	3	1	4	8	Good Quality
14	3	2	4	9	Good Quality
15	3	3	2	8	Good Quality
16	3	2	2	7	Medium Quality
17	3	2	2	7	Medium Quality
18	3	2	2	7	Medium Quality
19	3	3	4	10	Elite (High Quality)
20	3	2	4	9	Good Quality
21	3	2	2	7	Medium Quality
22	3	2	4	9	Good Quality
23	3	2	4	9	Good Quality
24	3	2	4	9	Good Quality
25	3	3	4	10	Elite (High Quality)
26	3	3	2	8	Good Quality
27	3	3	2	8	Good Quality
28	3	2	2	7	Medium Quality
29	3	2	4	9	Good Quality
30	3	2	2	7	Medium Quality
31	3	2	4	9	Good Quality
32	3	2	2	7	Medium Quality
33	3	2	4	9	Good Quality
34	3	2	4	9	Good Quality
35	3	2	4	9	Good Quality
36	3	3	4	10	Elite (High Quality)
37	3	2	4	9	Good Quality
38	3	2	4	9	Good Quality
39	3	2	2	7	Medium Quality
40	3	2	4	9	Good Quality
41	3	2	4	9	Good Quality
42	3	2	4	9	Good Quality
43	3	1	4	8	Good Quality
44	3	2	4	9	Good Quality
45	3	2	4	9	Good Quality
46	3	2	4	9	Good Quality
47	3	2	2	7	Medium Quality
48	3	2	2	7	Medium Quality
49	3	3	2	8	Good Quality
50	3	1	4	8	Good Quality
51	3	3	4	10	Elite (High Quality)
52	3	3	4	10	Elite (High Quality)
53	3	3	4	10	Elite (High Quality)
54	3	3	4	10	Elite (High Quality)
55	3	3	4	10	Elite (High Quality)
56	3	3	4	10	Elite (High Quality)
57	3	2	4	9	Good Quality
58	3	3	4	10	Elite (High Quality)
59	3	3	4	10	Elite (High Quality)
60	3	3	4	10	Elite (High Quality)
61	3	3	4	10	Elite (High Quality)
62	3	3	4	10	Elite (High Quality)
63	3	3	4	10	Elite (High Quality)
64	3	3	4	10	Elite (High Quality)
65	3	3	4	10	Elite (High Quality)
66	3	3	4	10	Elite (High Quality)
67	3	3	2	8	Good Quality
68	3	3	4	10	Elite (High Quality)
69	3	1	2	6	Medium Quality
70	3	3	2	8	Good Quality
71	3	2	2	7	Medium Quality
72	3	3	2	8	Good Quality
73	3	3	2	8	Good Quality
74	3	3	2	8	Good Quality
75	3	1	2	6	Medium Quality
76	3	3	2	8	Good Quality
77	3	2	2	7	Medium Quality
78	3	3	2	8	Good Quality
79	1	3	4	8	Good Quality
80	1	1	4	6	Medium Quality
81	3	3	4	10	Elite (High Quality)
82	3	3	4	10	Elite (High Quality)
83	3	3	4	10	Elite (High Quality)
84	3	3	4	10	Elite (High Quality)
85	3	3	4	10	Elite (High Quality)
86	3	2	4	9	Good Quality
87	3	3	4	10	Elite (High Quality)
88	3	2	4	9	Good Quality
89	3	3	4	10	Elite (High Quality)
90	3	2	4	9	Good Quality
91	3	2	4	9	Good Quality
92	3	2	4	9	Good Quality
93	3	2	4	9	Good Quality
94	3	2	4	9	Good Quality
95	1	2	4	7	Medium Quality
96	3	2	4	9	Good Quality
97	3	2	4	9	Good Quality
98	3	2	2	7	Medium Quality
99	3	3	4	10	Elite (High Quality)
100	3	3	4	10	Elite (High Quality)

**Table 8:** Summary of AMOVA for partitioning the genetic variance in 100 wheat genotypes distributed in four clusters generated via Hierarchical Cluster Analysis (Ward.D2 linkage and Jaccard distance) using 8 associated polymorphic markers at the *Glu-1* locus.

Source of Variation	df	Sum Sq	Mean Sq
Between samples	3	98.85	32.95 **
Within samples	96	173.09	1.80
Total	99	271.95	2.74

** Significant at *p* = 0.01.

## Data Availability

The raw data supporting the conclusions of this article will be made available by the authors on request.

## Supplementary Materials

The following supporting information can be downloaded at: https://www.mdpi.com/article/10.3390/plants15192965/s1. Table S1: Detailed information on 100 genotypes included in the study. Table S2: Mean values for 17 traits recorded on 100 wheat genotypes.

## Abbreviations

The following abbreviations are used in this manuscript:

DH	Days to heading
DM	Days to maturity
PHT	Plant height
PL	Peduncle length
SPKL	Spike length
SPKLTPS	Spikelets per spike
SPKWT	Single spike weight
GWTPSPK	Grain weight per spike
KPS	Kernel per spike
GY	Grain yield
TKW	1000-kernel weight
TWT	Test weight
GDI	Gluten deformation index
HMW-GS	High-molecular-weight glutenin subunits
PIC	Polymorphic information content
He	Expected heterozygosity
Ne	Number of alleles
I	Shannon’s information index
