# Supplementary material for: Phenotypic and Molecular Characterization of Winter Wheat Genotypes for Grain Yield, Quality Traits, and HMW Glutenin Subunits
Source: Plants (Basel). 2026 Sep 29;15(19):2965. doi: 10.3390/plants15192965 (PMC13666723; doi:10.3390/plants15192965)
Supplement: Supplementary file 1 [file plants-15-02965-s001.zip › Table_S1_Pedigree_of_the_100_genotypes_included_in_the_study.pdf]

Table S1. Information on the 100 winter wheat genotypes evaluated in 2020–2021 and 2021–2022 crop growing seasons in Kibray, Uzbekistan

Genotype number	Selection or name	Pedigree	Origin
1	27FAWWON-IR-9	Kauz*2/4/Car//Kal/BB/3/Nac/5/Kauz/6/Milan/7/Tilhi/8/4Won-Ir-257/5/Ymh/Hys//Hys/Tur3055/3/Dga/4/Vpm/Mos	IWWIP-Turkey#
2	27FAWWON-IR-12	UN-9/6/Attila/*Bcn//Bav92/3/Tilhi/5/Bav92/3/Prl/Sara//Tsi/Vee#5/4/Croc_1/Ae.squarrosa(224)//2*Opata	IWWIP-Turkey
3	27FAWWON-IR-13	UN-9/6/Attila/*Bcn//Bav92/3/Tilhi/5/Bav92/3/PRL/Sara//Tsi/Vee#5/4/Croc_1/Ae.squarrosa(224)//2*Opata	IWWIP-Turkey
4	27FAWWON-IR-14	UN-9/6/Attila/*Bcn//Bav92/3/Tilhi/5/Bav92/3/PRL/Sara//Tsi/Vee#5/4/Croc_1/Ae.squarrosa(224)//2*Opata	IWWIP-Turkey
5	27FAWWON-IR-25	Daphan//Pastor/Milan	IWWIP-Turkey
6	27FAWWON-IR-31	00*0100-51/3/*T. dicoccon* PI94625/*Ae. squarrosa*(372)//3*Pastor	IWWIP-Turkey
7	27FAWWON-IR-38	FD07072/4/Pfau/Milan/3/Babax/LR42//Babax/5/Pfau/Seri.1B//Amad/3/Waxwing	IWWIP-Turkey
8	27FAWWON-IR-39	Mercato//92.001E7.32.5/Slvs	IWWIP-Turkey
9	27FAWWON-IR-41	Mercato//92.001E7.32.5/Slvs	IWWIP-Turkey
10	27FAWWON-IR-45	Dorade-5/3/SHI#4414/Crows"//Gksagvari/CA8055	IWWIP-Turkey
11	27FAWWON-IR-46	Dorade-5/3/SHI#4414/Crows"//Gksagvari/CA8055	IWWIP-Turkey
12	27FAWWON-IR-53	Zander-44/4/338-K1-1//Anb/Buc/3/GS50A/5/K1329A4	IWWIP-Turkey
13	27FAWWON-IR-58	Sabalan//Trego/Btysib	IWWIP-Turkey
14	27FAWWON-IR-69	Aisberg/AE.Squarrosa(369)//Demir	IWWIP-Turkey

15	27FAWWON-IR-70	Aisberg/Ae.squarrosa(369)//Demir	IWWIP-Turkey
16	27FAWWON-IR-73	MV-Kolompos/4/Mahon Demias/3/Him/Cndr//CA8055	IWWIP-Turkey
17	27FAWWON-IR-75	PP346/Novazvezda/5/Cupra-1/3/Croc1/AE.Squarrosa (224)//2*Opata/4/Pantheon/6/Cupra-1/3/Croc1/Ae. squarrosa (224)//2*Opata/4/Pantheon	IWWIP-Turkey
18	27FAWWON-IR-76	53/3/ABL/1113//K92/4/Jag/5/KS89180B/6/PBW343*2/Kukuna*2//YAnac	IWWIP-Turkey
19	27FAWWON-IR-81	KS82142/Pastor/3/Attila*2/PBW65*2//Yanac	IWWIP-Turkey
20	27FAWWON-IR-84	Areso/Roelfs F2007	IWWIP-Turkey
21	27FAWWON-IR-86	Tirchmir1//71ST2959/Crow/3/KS 92 WGRC24/4/Whear/Sokoll	IWWIP-Turkey
22	27FAWWON-IR-91	TAM200/Kauz/7/91-142A61/3/F35.70/MO73//1D13.1/Mlt/6/PI/ MZ//Cno67/3/Lfn/4/Ant/5/Attila	IWWIP-Turkey
23	27FAWWON-IR-103	2CA542C/Skorospelka/Neuzuct/3/Nac76	IWWIP-Turkey
24	27FAWWON-IR-110	Konkurent	Russia
25	27FAWWON-IR-123	(Attila*2/Esda//Mason)/(HBK0935-7-4/Betty's')//KS91W047	IWWIP-Turkey
26	27FAWWON-IR-124	OK01307/Duster//OK06822W	IWWIP-Turkey
27	27FAWWON-IR-125	Duster/Billings	USA-OSU
28	27FAWWON-IR-136	ORH010085/2*Agripro Palomino-1	IWWIP-Turkey
29	27FAWWON-IR-169	Onearly_S-148/ES99S2-14	IWWIP-Turkey
30	27FAWWON-IR-142	KS13DH002722	USA-KSU
31	27FAWWON-IR-150	KS13DH0045-84	USA-KSU
32	27FAWWON-IR-153	KS14DH0012-12	USA-KSU

33	27FAWWON-IR-157	KS100196K-2	USA-KSU
34	27FAWWON-IR-172	Bojana	Bulgaria
35	27FAWWON-IR-199	3971-6 (Bul)/6/Sideral/3/Bau/Lira//Kauz/5/Lov26//Lfn/Sdy(ES84-24)/3/Seri/4/Seri	IWWIP-Turkey
36	27FAWWON-IR-200	3971-6 (BUL)/6/Sideral/3/Bau/Lira//Kauz/5/Lov26//Lfn/Sdy(ES84-24)/3/Seri/4/Seri	IWWIP-Turkey
37	20AWYT-IR-20	ID800994.W/Vee/4/Kvz/Cut75/3/Ymh//61.1523/Drc/6/Akul/5/Gov/AZ//Mus/3/Dodo/4/Bow	IWWIP-Morocco
38	20AWYT-IR-30	Grk79//Inqalab 91*2/Tukuru	IWWIP-Morocco
39	Rapsudiya	Commercial cultivar (Agri/Nakozari F76)	Serbia
40	20AWYT-IR-49	Cadet/6/Yumai13/5/Nai60/3/14.53/Odin//CI13441/Canon/7/Zander//Attila/3*Bcn	IWWIP-Morocco
41	20AWYT-IR-52	Cadet/6/Yumai13/5/Nai60/3/14.53/Odin//CI13441/Canon/7/Zander//Attila/3*Bcn	IWWIP-Morocco
42	20AWYT-IR-54	Cadet/6/Yumai13/5/Nai60/3/14.53/Odin//CI13441/Canon/7/Zander//Attila/3*Bcn	IWWIP-Morocco
43	20AWYT-IR-59	Kleiber/2*FL80//Donsk.Poluk./3/KS82W409/Stephens//Hatusha/4/Bezostaya	IWWIP-Morocco
44	20AWYT-IR-75	Shii#4414/Crows"//GK Sagvari/CA8055/3/Aziz	IWWIP-Morocco
45	20AWYT-IR-78	Shi#4414/Crows"//GK Sagvari/CA8055/3/Aziz	IWWIP-Morocco
46	20AWYT-IR-85	Shi#4414/Crows"//GK Sagvari/CA8055/6/4Won-Ir-257/5/Ymh/Hys//Hys/Tur3055/3/Dga /4/Vpm/Mos	IWWIP-Morocco
47	20AWYT-IR-87	Shi#4414/Crows"//GK Sagvari/CA8055/6/4Won-Ir-257/5/Ymh/Hys//Hys/Tur3055/3/Dga /4/Vpm/Mos	IWWIP-Morocco
48	Graf	Commercial cultivar (Lutescens2184 h 31/Fortuna)	Russia
49	Step	Commercial cultivar (L.369-93k14/Back Palenque)	Russia
50	20AWYT-IR-113	Zander//Attila/3*Bcn/3/Sadovska Belija/Pehlivan	IWWIP-Morocco
51	20AWYT-IR-123	Zander//Attila/3*Bcn/3/Pantheon/Bluegil-2	IWWIP-Morocco

52	20AWYT-IR-126	Zander//Attila/3*Bcn/3/MV Dalma/Lagos-6	IWWIP-Morocco
53	Kunak	Commercial cultivar (Pedigree unknown)	Russia
54	20AWYT-IR-141	Psk/Nac//Sabalan/3/Gun91/Mnch/5/Rsk/CA8055//Cham6/4/Nwt/3/Tast/Sprw//Taw12399.75	IWWIP-Morocco
55	Lebed	Commercial cultivar (Yashkulyanka/90–4T18)	Russia
56	20AWYT-IR-298	4Won-Ir-257/5/Ymh/Hys//Hys/Tur3055/3/Dga /4/ Vpm /Mos/6/Cham-8	IWWIP-Morocco
57	20AWYT-IR-322	Haitham 18/8/Trap#1/Yaco/3/Kauz2/Trap//Kauz/6/SN64//Ske/*Ane/3/SX/4/Bez/5/Jun/7/Shark-1	IWWIP-Morocco
58	20AWYT-IR-324	Gul96/Shark-1/6/Ymh/Hys//Hys/Tur3055/3/Dga/4/Vpm/Mos/5/5/Tam200/Kauz/7/Jagger 'Sib' /3/ Lagos-7//Guimatli 2/17	IWWIP-Morocco
59	20AWYT-IR-327	Zander//Attila/3*Bcn/6/Bez//Cno/Gll/3/Vrz/4/Bjn C47/5/Passarinho/ 7/4Wonir-257/5/Ymh/Hys//Hys/Tur3055/ 3/Dga /4/Vpm /Mos	IWWIP-Morocco
60	20AWYT-IR-331	Zander//Attila/3*Bcn/6/Bez//Cno/Gll/3/Vrz/4/Bjn C47/5/Passarinho/ 7/4Wonir-257/5/Ymh/Hys//Hys/Tur3055/ 3/Dga /4/Vpm /Mos	IWWIP-Morocco
61	20AWYT-IR-332	Zander//Attila/3*Bcn/6/Bez//Cno/Gll/3/Vrz/4/Bjn C47/5/Passarinho/ 7/4Wonir-257/5/Ymh/Hys//Hys/Tur3055/ 3/Dga /4/Vpm /Mos	IWWIP-Morocco
62	20AWYT-IR-336	Psk/Nac//Sabalan/3/Gun91/Mnch/4/AUS4930 6.5/Pastor /5/Dorade-5//KS82117/Mlt	IWWIP-Morocco
63	20AWYT-IR-338	Psk/Nac//Sabalan/3/Gun91/Mnch/4/AUS4930 6.5/Pastor /5/Dorade-5//KS82117/Mlt	IWWIP-Morocco
64	20AWYT-IR-352	JI5418/Maras/4/885K4.1//Mng/Sdv1/3/1D13.1/Mlt/5/Seri 82/ Shuha's'//Dovin-2	IWWIP-Morocco
65	23IWWYT-IR-9808	Patwin Yr15/L 4224 K 12	IWWIP-Turkey
66	20IWWYT-IR-9812	Nikifor/Kroshka	IWWIP-Turkey
67	23IWWYT-IR-9815	Victorya/4/WblL1/3/Tsi/Vee#5//Kauz/5/Vorona//Milan/Sha7	IWWIP-

		/3/MV17	Turkey
68	23IWWYT-IR-9816	Nemura/Crdn//78014.40/3/Attila/Bav92//Pastor/4/Gondvana	IWWIP-Turkey
69	23IWWYT-IR-9818	MV22-2000/3/Agri/Nac//Kauz	IWWIP-Turkey
70	23IWWYT-IR-9820	Patwin Yr5/5/Tam105/3/NE70654/Bby//Bow"S"/4/ Century*3/Ta2450	IWWIP-Turkey
71	23IWWYT-IR-9821	Patwin Yr5/5/Tam 105/3/NE70654/Bby//Bow"S"/4/ Century*3/TA2450	IWWIP-Turkey
72	23IWWYT-IR-9825	Croc_1/Ae.squarrosa (205)//Kauz/3/Lufer/4/KS97P0630-4-5/CM95560//X920879-C15-1/3/X84W063-9-18/U1324-25-1-4/5/KS82142/Pastor	IWWIP-Turkey
73	23IWWYT-IR-9829	OK81306/Star's'	IWWIP-Turkey
74	23IWWYT-IR-9841	KS031009K-4/KS060142-K-4	USA-KSU
75	23IWWYT-IR-9842	KS040477K-12/Gallagher	USA-KSU
76	23IWWYT-IR-9843	KS031009K-4/KS060285-M-1	USA-KSU
77	23IWWYT-IR-9846	OK14319	USA-OSU
78	23IWWYT-IR-9847	OK12D22004-016	USA-OSU
79	Chillaki	Commercial cultivar (Genrumil/Yuktina)	Uzbekistan
80	Grom	Commercial cultivar (2919K3/S1171-95)	Russia
81	Bezostaya 100	Commercial cultivar (Lutescens3415k8-6-4/Lutescens198-93k80)	Russia
82	Antonina	Commercial cultivar (Lutescence94-409A656-2/Line Otzim)	Russia
83	Zimnitsa	Commercial cultivar (Zimorodok151/Lutesence1221K7214)	Russia
84	ASR	Commercial cultivar (Kroshka/94-409A627)	Uzbekistan
85	Zvezda	Commercial cultivar (Pedigree unknown)	Uzbekistan
86	Zamin 1	Commercial cultivar (Sanzar-8/Parazuki)	Uzbekistan
87	Krasnodar 99	Commercial cultivar (KN2665G1023/KN4695h449//KN2621h24-82)	Russia
88	Durdona	Commercial cultivar (570t189-160//9t 60-1-69/L.2739h102-	Uzbekistan

		4)	
89	Gozgon	Commercial cultivar (Agri/Bjy//Vee/3/Akula/4/F10S-1)	Uzbekistan
90	Semurug	Commercial cultivar (Unumli bugday/K429696)	Uzbekistan
91	N29/2016	Pedigree unknown	Uzbekistan
92	N83/2016	Pedigree unknown	Uzbekistan
93	КП-84/2017	Pedigree unknown	Uzbekistan
94	КП-85/2017	Pedigree unknown	Uzbekistan
95	КП-125/2017	Pedigree unknown	Uzbekistan
96	КП-153/2017	Pedigree unknown	Uzbekistan
97	КП-183/2017	Pedigree unknown	Uzbekistan
98	КП-184/2017	Pedigree unknown	Uzbekistan
99	КП-197/2017	Pedigree unknown	Uzbekistan
100	Pahlavon	Commercial cultivar (Sanzar85/K95137)	Uzbekistan

#IWWIP = International Winter Wheat Improvement Program; KSU = Kansas State University, USA; OSU = Oklahoma State University, USA
