# Supplementary material for: Phenotypic and Molecular Characterization of Winter Wheat Genotypes for Grain Yield, Quality Traits, and HMW Glutenin Subunits
Source: Plants (Basel). 2026 Sep 29;15(19):2965. doi: 10.3390/plants15192965 (PMC13666723; doi:10.3390/plants15192965)
Supplement: Supplementary file 1 [file plants-15-02965-s001.zip › Table_S2_Mean_Values_for_17_traits_in_a_study_of_100_winter_wheat.pdf]

Table S2. Mean values for different traits in 100 winter wheat genotypes evaluated in Kibray, Uzbekistan in 2021 and 2022

Genotype	DH#	DM	PHT	PL	SPKL	SPKLTPS	SPKWT	GWTPSPK	KPS	GY	TKW	TWT	Protein#	Gluten£	GDI¥	Sedimentation¶	Vitreousness
	days	days	cm	cm	cm	per spike	g	g	per spike	t/ha	g	g/l	%	%	%	ml	%
1	160	202	101	37	11	18	3.10	2.45	50	5.22	40.7*	756ns	15.3*	30.4ns	96.8	38.8	85
2	156	205	99	39	11	19	3.14	2.34	55	6.89 *	36.1 ns	781*	14.9ns	29.9ns	81.3	49.5	71
3	159	205	99	39	9	17	2.32	1.71	44	6.51 *	37.8*	776*	15.7*	32.9ns	83.5	42.7	84
4	158	206	104	41	10	18	2.26	1.69	46	5.43	33.7	779*	14.9ns	30.1ns	82.4	41.8	61
5	159	204	102	34	10	19	2.60	2.05	47	6.22ns	41.3*	772*	15.3*	30.0ns	88.0	34.8	76
6	162	207	95	36	9	16	2.38	1.87	42	5.35	37.2*	793*	16.1*	34.9*	89.3	39.0	70
7	163	206	104	36	10	20	2.67	2.03	51	6.06ns	35.2 ns	769*	15.0ns	28.4	104.4	31.3	69
8	164	211	96	35	9	17	2.41	1.94	49	6.13ns	36.2 ns	750ns	14.0	26.6	81.8	37.3	59
9	165	209	100	36	10	17	2.44	1.96	48	6.76*	39.6*	752ns	13.0	26.4	80.6	40.2	61
10	160	207	104	42	11	18	2.34	1.79	41	5.57	37.3*	740	14.5ns	30.5ns	89.0	37.0	63
11	161	206	106	40	10	18	2.25	1.72	43	6.89*	38.0*	765*	15.0ns	30.1ns	94.8	34.3	69
12	166	207	113	44	10	18	2.41	1.83	42	5.77ns	38.9*	764*	17.2*	36.8*	86.1	37.3	69
13	163	208	116	43	11	19	2.47	1.91	45	6.34ns	36.9*	740	16.1*	31.2ns	88.9	32.0	67
14	163	205	99	37	10	18	2.30	1.86	40	5.74ns	38.5*	767*	15.5*	32.6ns	78.2	42.7	68
15	162	206	103	38	11	18	2.43	1.92	41	6.20ns	37.5*	762ns	16.2*	35.2*	87.9	45.2	65
16	162	210	101	38	10	19	2.53	1.97	45	6.57*	40.4*	731	15.7*	30.9ns	103.4	29.5	62
17	161	204	110	35	9	19	2.66	2.24	54	5.19	34.9 ns	704	15.6*	33.0ns	79.8	37.5	57
18	162	204	103	37	10	18	2.28	1.74	46	4.93	34.9 ns	767*	14.7ns	30.6ns	86.2	36.8	65
19	158	207	98	34	10	20	2.77	2.04	52	6.10ns	33.1	764*	15.0ns	31.6ns	91.3	42.0	58
20	159	204	98	30	10	17	2.65	2.10	50	6.35ns	33.4	756 ns	15.7*	32.3ns	84.4	43.0	78
21	159	208	94	34	9	17	2.46	1.88	47	6.44*	36.9*	751 ns	15.4*	35.5*	82.5	37.8	75
22	163	207	104	41	10	19	2.78	2.16	52	4.98	36.2 ns	778*	14.3ns	30.2ns	103.6	42.2	75
23	162	205	110	39	10	19	2.78	2.17	50	4.85	35.8 ns	665	15.7*	30.9ns	93.1	30.5	60

24	164	207	79	33	8	18	1.76	1.50	36	5.64	34.5 ns	747 ns	15.7*	31.4ns	73.9	36.8	70
25	165	206	101	39	10	19	2.04	1.66	38	4.93	39.4*	762 ns	16.7*	36.4*	81.8	35.7	77
26	163	206	105	36	10	19	2.45	1.78	47	4.24	35.6 ns	751 ns	14.9ns	30.5ns	102.9	39.7	61
27	162	205	94	36	9	16	2.44	1.85	44	5.85	35.2 ns	759 ns	14.9ns	32.0ns	76.9	40.8	70
28	166	208	95	33	10	18	2.55	1.98	48	4.45	37.5*	731	16.6*	30.4ns	95.2	42.0	77
29	166	206	98	37	9	18	2.36	1.80	41	4.75	37.1*	736	15.6*	33.3ns	96.4	31.2	59
30	161	203	92	35	9	17	2.25	1.73	39	6.18ns	35.5 ns	786*	16.6*	35.8*	88.6	37.5	67
31	163	206	91	33	9	18	1.86	1.50	40	5.94	31.9	744 ns	15.1ns	31.3ns	86.8	39.7	65
32	162	205	98	34	11	19	3.02	2.48	58	4.44	34.9 ns	717	15.0ns	31.3ns	83.3	37.8	59
33	164	204	102	37	10	18	1.86	1.38	44	6.16ns	31.5	744 ns	14.4ns	30.2ns	79.1	38.0	67
34	165	209	110	41	10	20	2.51	2.00	45	5.56	43.0*	747 ns	16.7*	35.7*	87.2	41.2	64
35	164	209	96	38	9	20	3.20	2.50	58	6.37*	38.2*	751 ns	14.9ns	26.8	109.7	31.8	76
36	164	209	96	40	9	20	3.28	2.58	66	6.09ns	36.8*	719	14.6ns	29.5ns	102.1	32.0	72
37	163	207	109	38	11	19	2.68	2.03	55	6.24ns	36.4*	735	15.3*	32.5ns	93.2	29.3	58
38	157	203	95	36	10	17	2.77	2.12	46	4.11	33.9	737	15.3*	32.7ns	83.3	38.0	66
39	160	206	97	37	10	20	2.57	2.00	50	5.74	33.9	760 ns	14.3ns	31.1ns	79.2	40.5	74
40	160	205	111	39	11	17	1.90	1.47	47	5.28	34.6 ns	754 ns	15.2*	33.1ns	85.8	35.2	67
41	159	204	111	41	9	18	2.33	1.98	47	5.97ns	38.3*	749 ns	15.0ns	30.4ns	104.7	29.0	70
42	159	203	113	42	11	18	3.01	2.27	51	6.64*	38.3*	785*	15.4*	32.6ns	79.9	45.2	64
43	162	205	95	38	10	17	2.59	2.07	47	5.87	39.1*	765*	15.3*	31.8ns	87.1	39.5	74
44	160	205	104	42	12	19	2.62	2.13	49	6.50*	39.3*	774*	15.3*	31.5ns	83.8	33.8	75
45	159	205	99	41	12	22	2.52	1.92	50	4.62	36.1 ns	774*	15.6*	31.6ns	83.4	44.0	75
46	159	203	92	35	10	19	2.62	2.01	47	5.36	36.0 ns	753 ns	14.8ns	32.5ns	91.2	38.0	66
47	159	207	108	40	9	17	2.48	1.97	47	6.45*	41.4*	788*	16.2*	35.9*	93.4	37.5	71
48	165	209	82	34	9	18	2.05	1.65	46	6.07ns	30.4	707	14.8ns	30.3ns	85.7	36.8	68
49	165	207	83	32	9	17	2.22	1.74	47	5.34	32.2	714	14.9ns	31.0ns	88.0	39.8	59
50	161	209	105	41	10	18	2.82	2.26	57	6.08ns	35.1 ns	777*	14.2ns	29.4ns	92.8	39.3	70
51	160	205	109	38	12	21	2.54	2.06	53	6.31ns	35.9 ns	781*	15.0ns	31.8ns	87.5	41.3	79

52	163	206	104	37	10	19	2.05	1.62	44	6.72*	34.4	762 ns	15.5*	31.8ns	93.7	29.0	77
53	163	206	98	38	12	21	2.39	1.80	46	5.17	37.4*	736	14.5ns	29.9ns	97.2	41.2	63
54	166	208	112	44	11	20	2.55	1.79	42	4.60	36.1 ns	721	16.0*	28.3	110.6	26.8	78
55	162	205	101	37	12	20	2.46	1.95	51	6.78*	34.3	763 ns	15.6*	32.2ns	87.9	38.5	66
56	159	207	95	38	10	18	2.56	1.87	46	4.94	34.4	779*	16.2*	34.2*	85.9	40.2	87
57	159	206	103	41	11	20	2.63	2.04	49	5.42	34.0	745 ns	15.7*	33.2ns	85.7	39.7	79
58	162	206	101	42	12	19	3.41	2.55	52	5.24	39.5*	757 ns	16.2*	33.4ns	84.2	40.7	67
59	158	210	92	33	10	18	2.88	2.37	53	7.45*	40.2*	772*	14.1 ns	29.6ns	86.1	35.7	72
60	159	206	90	32	9	18	2.62	2.03	55	5.03	36.9*	784*	14.3ns	31.7ns	80.4	36.7	76
61	156	203	105	36	10	17	2.93	2.33	48	7.22*	40.9*	800*	13.9	30.0ns	95.0	35.7	82
62	163	207	103	40	10	18	2.70	2.19	53	5.18	34.4	787*	15.8*	33.0ns	92.0	40.7	77
63	161	204	93	37	10	18	2.17	1.65	50	5.56	31.5	750 ns	14.5ns	25.9	111.2	36.2	77
64	159	204	109	43	10	18	2.54	1.96	41	4.29	45.9*	727	14.4ns	32.3ns	83.9	44.5	63
65	162	207	99	34	12	18	2.29	1.86	43	5.76	37.4*	696	16.2*	32.6ns	83.5	35.7	64
66	166	209	103	41	10	19	2.15	1.67	44	6.70*	36.0 ns	767*	14.6ns	28.5	87.4	42.7	62
67	162	207	105	39	11	21	2.52	1.99	51	6.00ns	34.8	766*	15.1ns	31.0ns	83.1	39.7	56
68	158	205	102	37	10	16	2.37	1.87	44	6.79*	33.4	741ns	14.1	29.5ns	81.3	34.5	68
69	162	204	100	42	9	19	2.00	1.57	44	4.76	34.0	758 ns	15.2*	31.8ns	95.2	27.8	84
70	160	203	97	33	11	19	2.21	1.68	47	6.10ns	32.1	749 ns	15.3*	29.5ns	91.8	37.0	62
71	160	204	96	33	11	20	2.43	1.90	48	4.89	37.1*	751 ns	15.8*	33.4ns	78.6	47.2	74
72	161	207	102	36	10	19	2.20	1.60	40	6.25ns	38.6*	792*	15.4*	28.9	113.6	26.0	71
73	161	210	108	40	11	19	3.07	2.32	50	6.54*	39.4*	749 ns	15.1ns	30.9ns	78.4	42.7	62
74	162	206	93	31	9	17	2.29	1.84	47	6.00ns	33.4	753 ns	16.0*	30.2ns	83.8	45.0	66
75	165	208	90	32	8	17	2.02	1.57	46	6.37*	31.6	757 ns	14.6ns	30.1ns	87.1	45.3	61
76	162	204	92	33	8	15	2.14	1.68	44	6.27ns	33.7	784*	15.9*	31.2ns	76.2	45.2	71
77	163	209	99	35	9	16	1.95	1.52	38	7.09*	34.8 ns	788*	14.8ns	28.3	81.3	38.3	62
78	160	206	95	34	8	16	2.13	1.70	43	6.42*	36.0 ns	778*	14.2ns	29.4ns	79.2	41.3	73
79	154	200	84	33	9	18	2.49	2.00	48	5.31	36.7*	752 ns	14.4ns	31.8ns	92.5	37.2	65
80	164	211	98	35	10	20	2.37	1.91	49	6.16	35.4	752	14.6	31.5	80.2	38.0	63
81	163	208	89	38	10	22	2.15	1.66	45	4.99	37.2*	758 ns	16.1*	32.5ns	88.2	41.8	83

82	166	212	99	37	12	21	3.05	2.32	56	5.53	38.1*	747 ns	15.5*	31.7ns	94.4	39.0	66
83	163	205	98	39	10	19	2.56	2.11	50	5.09	43.4*	768*	14.1	29.7ns	74.3	38.7	70
84	161	205	99	38	11	20	2.89	2.37	51	6.85*	43.4*	764*	14.1	27.0	89.8	44.3	69
85	159	205	96	33	10	18	2.13	1.69	41	6.74*	36.0 ns	770*	16.1*	31.1ns	92.5	35.2	66
86	159	206	98	37	11	18	2.85	2.19	52	5.78	39.5*	734	14.8ns	26.8	95.2	38.2	73
87	164	208	93	36	10	21	2.37	1.98	47	5.95	37.5*	761 ns	14.5ns	30.0ns	86.3	38.3	67
88	163	209	109	43	11	21	2.34	1.87	47	6.26ns	34.2	781*	15.2*	32.2ns	91.0	38.3	72
89	162	206	103	39	9	17	2.70	1.94	48	6.21ns	37.1*	685	15.6*	30.2ns	86.2	41.7	65
90	158	206	93	34	11	19	2.82	2.20	56	6.11ns	41.7*	739	13.5	28.5	79.4	45.2	59
91	157	207	113	36	10	19	3.03	2.24	47	5.19	44.0*	724	15.4*	28.0	100.1	28.3	76
92	160	208	96	39	11	18	2.93	2.17	47	6.01ns	41.8*	744 ns	14.1	29.1	94.5	37.7	59
93	160	210	96	36	12	19	3.46	2.49	60	5.94	39.3*	736	14.0	27.8	97.4	36.5	59
94	159	205	98	36	12	18	2.75	2.13	47	4.93	38.2*	775*	14.9ns	28.9	91.4	37.3	74
95	161	205	103	39	10	16	2.77	2.04	48	6.27ns	40.8*	783*	14.0	30.9ns	87.0	43.8	73
96	160	206	95	35	10	18	2.75	2.10	46	5.27	40.1*	781*	14.3ns	30.0ns	91.5	36.7	73
97	162	206	99	40	10	17	2.64	2.09	46	6.60*	37.5*	787*	14.4ns	30.1ns	87.3	48.7	73
98	157	205	96	34	10	18	2.72	2.04	47	6.16ns	38.3*	772*	14.2ns	30.1ns	83.6	45.3	70
99	157	205	104	40	11	19	2.45	1.89	53	5.03	35.2 ns	770*	14.8ns	29.4ns	89.6	37.3	71
100	159	205	116	46	10	20	2.54	1.93	42	5.75	40.3*	786*	15.9*	31.7ns	92.7	37.8	63
Mean	161	206	99.95	37	10	18	2.52	1.96	48	5.81	36.9	756	15.1	31.0	88.8	38.3	69
$LSD_{0.05}$	1.5	1.7	6.9	3	1	1.3	0.15	0.09	1.8	0.20	0.9	11	0.5	2.2	5.4	2.3	3.8
CV (%)	0.85	1	6	8	6.12	6.54	5.50	4.33	3.2	3.04	2.1	1.3	3.2	6.3	5.3	5.2	4.9
Min	154	200	79	30	8	15	1.8	1.4	36	4.11	30	665	13.0	25.9	74	26	56
Max	166	212	116	46	12	22	3.5	2.6	66	7.45	46	800	17.2	36.8	114	50	87

#DH = Days to heading, DM = Days to maturity, PHT = Plant height, PL = Peduncle length, SPKL = Spike length, SPKPSP = Spikelet per spike, SPKW = Single spike weight, GWTPSP = Grain weight per spike, KPS = Kernel per spike, GY = Grain yield, TKW = 1000-kernel weight, TWT = Test weight, GDI = Gluten deformation index

[#]Minimum values for Grade I and II wheat for Uzbekistan are respectively 14 and 12.5% protein and 32 and 28% gluten.

[¥]GDI value weak, normal and strong wheat are <30%, 30 to 80% and >80%, respectively.

[¶]Sedimentation different grades of wheat are poor <20 ml, satisfactory 20 to 30 ml, good 31 to 50 ml and very good >50 ml.

[≠]Vitreous value for Grade I and II wheat in Uzbekistan is >60%.
